# Reducing biomass recalcitrance by heterologous expression of a bacterial peroxidase in tobacco (*Nicotiana benthamiana*)

**DOI:** 10.1038/s41598-017-16909-x

**Published:** 2017-12-06

**Authors:** Ayalew Ligaba-Osena, Bertrand Hankoua, Kay DiMarco, Robert Pace, Mark Crocker, Jesse McAtee, Nivedita Nagachar, Ming Tien, Tom L. Richard

**Affiliations:** 1 College of Agriculture and Related Sciences, Delaware State University, 1200 N DuPont Highway, Dover, DE 19901 USA; 2 Agricultural and Biological Engineering, Pennsylvania State University, 111 Research Unit A, University Park, Pennsylvania, PA 16802 USA; 3 Center for Applied Energy Research, University of Kentucky, 2540 Research Park Drive, Lexington, KY 40511 USA; 4 Department of Chemistry and Biochemistry, University of Delaware, Newark, DE 19716 USA; 5 Department of Biochemistry and Molecular Biology, Pennsylvania State University, 305 South Frear Laboratory, University Park, Pennsylvania, PA 16802 USA

## Abstract

Commercial scale production of biofuels from lignocellulosic feed stocks has been hampered by the resistance of plant cell walls to enzymatic conversion, primarily owing to lignin. This study investigated whether DypB, the lignin-degrading peroxidase from *Rodococcus jostii*, depolymerizes lignin and reduces recalcitrance in transgenic tobacco (*Nicotiana benthamiana*). The protein was targeted to the cytosol or the ER using ER-targeting and retention signal peptides. For each construct, five independent transgenic lines were characterized phenotypically and genotypically. Our findings reveal that expression of DypB in the cytosol and ER does not affect plant development. ER-targeting increased protein accumulation, and extracts from transgenic leaves showed higher activity on classic peroxidase substrates than the control. Intriguingly, *in situ* DypB activation and subsequent saccharification released nearly 200% more fermentable sugars from transgenic lines than controls, which were not explained by variation in initial structural and non-structural carbohydrates and lignin content. Pyrolysis-GC-MS analysis showed more reduction in the level of lignin associated pyrolysates in the transgenic lines than the control primarily when the enzyme is activated prior to pyrolysis, consistent with increased lignin degradation and improved saccharification. The findings reveal for the first time that accumulation and *in situ* activation of a peroxidase improves biomass digestibility.

## Introduction

Increasing concern over global climate change has necessitated the development of alternative and renewable energy sources. ‘First generation’ biofuels such as ethanol have been produced from starch and sugar-based raw materials including corn, sorghum, sugarcane and sugar beet in U.S., Brazil and the E.U. countries. However, production of biofuels from these feedstocks has raised public concerns due to competition for land, food and feed supplies. While lignocellulosic biomass-based ‘second generation’ biofuels are advancing rapidly^1–3^, the technologies required for large-scale, cost-effective conversion of lignocellulosic biomass to biofuels are still under development. The main challenge is biomass recalcitrance (*i*. *e*. the resistance of the plant cell wall to deconstruction) owing to the presence of lignin^4–7^. Lignin is a complex heterogeneous alkyl-aromatic polymer derived primarily from three hydroxycinnamyl alcohol monomers (p-coumaryl, coniferyl- and sinapyl-alcohols) via radical coupling, and occurs in tight association with polysaccharides cellulose and hemicellulose^4,8^. Lignin is the second most abundant biopolymer on Earth (after cellulose), comprising 15–30% dry weight of the lignocellulose component of plant cell walls, and consists of phenylpropanoid units linked via carbon-oxygen-carbon (C-O-C, ether) and carbon-carbon (C-C) bonds^9,10^. Many research laboratories are focused on developing commercially viable biomass pretreatment strategies to reduce or eliminate cell wall recalcitrance in order to increase enzyme accessibility and cellulose digestibility.

Over the last several decades, various pretreatment strategies have been developed including physical (mechanical disruption), chemical (dilute acid, alkali, solvents etc.) and physico-chemical (ammonia fiber or steam explosion) methods^3,11,12^. While these strategies can reduce biomass recalcitrance to some extent, they suffer from one or more challenges such as high energy demand, high chemical costs, formation of fermentation inhibitors, low sugar yield, generation of toxic compounds or reactor corrosion^3,12–14^. Biological pretreatment using ligninolytic microorganisms such as white, brown, and soft-rot fungi and bacteria is another approach that has been well studied^13,15–17^. This approach is believed to require lower operating costs, is relatively safe and environmentally friendly^3,13,15^, and may enable enzyme (laccases and lignin-peroxidase)-mediated detoxification of fermentation inhibitors^18,19^. However, there are techno-economic problems associated with biological treatment including low saccharification efficiency, the need for large pretreatment space, and careful optimization of microbial growth conditions^3,17^. In addition, most ligninolytic microorganisms hydrolyze hemicellulose and cellulose as well as lignin, and potential degradation of sugar polymers makes this approach less attractive commercially^17,20^. Indeed, despite tremendous efforts to develop commercially viable methods, pretreatment remains the most expensive unit operation in the conversion of lignocellulosic feedstocks to biofuels, accounting for nearly $0.30/gallon of ethanol produced^21^.

Several biotechnological and genetic approaches have been attempted to reduce biomass recalcitrance, but none have been utilized in the biofuel industry on a commercial scale thus far^22^. A number of glycoside hydrolase (GH) enzymes have been expressed *in planta* aimed at reducing the cost of enzyme production as compared to fungal sources^23–27^. While encouraging achievements have been reported, this method also suffers drawbacks including the need of the plants to produce a large amount of enzyme, thereby placing a metabolic burden on plants, increasing fertilizer inputs and the risk of undesirable effects on normal plant development, and requiring additional capital and operating costs^22^. Another interesting attempt to reduce biomass recalcitrance has involved manipulating the expression of genes and transcription factors that are involved in the lignin biosynthetic pathway. For example, antisense RNA-mediated downregulation of the shikimate hydroxycinnamoyl transferase (HCT) significantly reduces lignin content and improved cell wall digestibility in alfalfa (*Medicago sativa*)^5^. Likewise, overexpression of the transcription factor *PvMYB4* which regulates monolignol pathway genes resulted in reduced lignin content, and increased sugar release efficiency in transgenic switchgrass (*Panicum virgatum)* by approximately three-fold^28^. Similarly, ectopic overexpression of the maize non-coding small RNAs (miR156) in transgenic switchgrass^29^ has been shown to reduce lignin content and improve biomass saccharification efficiency with or without pretreatment.

Naturally, members of the *Basidiomycetes* fungi depolymerize lignin by using powerful oxidative enzymes^30–32^ such as lignin peroxidases (LiPs, EC 1.11.1.14)^33^, manganese peroxidases (MnPs, EC 1.11.1.13)^34^, versatile peroxidases (VPs, EC 1.11.1.16; that possess the structural-functional properties of LiPs and MnPs)^35^, and laccases (EC 1.10.3.2)^36^. While these enzymes are exclusively reported from fungi^37^, the ability to depolymerize lignin has also been documented in bacteria^38^ although the enzymology of bacterial lignin degradation was poorly understood until recently^32^. The first heme-containing peroxidase named DyP (dye-decolorizing peroxidase, EC1.11.1.19) was isolated from the fungus *Bjerkandera adusta* (initially described as *Geotrichum candidum*), and bore no homology to known peroxidases^39^. Subsequently, a number of genes belonging to the DyP-type peroxidases superfamily were identified from other fungi including *Termitomyces albuminosus* ^40^, *Marasmius scorodonius* ^41^, *Thanatephorus cucumeris* Dec 1^42^ and *Auricularia auricula-judae* ^43^. Over the last decade, several DyP-type proteins have also been isolated from a number of bacterial species including *E*. *coli* ^44^, *Rodococcus jostii* RHA1^45^, *Amycolatopsis* sp. 75iv2^46^, *Pseudomonas spp*.^47,48^ and *Bacillus subtilis* ^49^. The DyP-type peroxidases catalyze the oxidation of a range of substrates including synthetic dyes, non-phenolic methoxylated aromatics, lignin, β-carotene and Mn^2+^ ^50^. At least four phylogenetically distinct subfamilies with varying specificities towards substrates such as the anthraquinone compound (reactive blue) have been identified^38,51^. The DyPs from bacteria belong to the A, B and C subfamilies, while the fungal enzymes fall in the D-subfamily.

Bioinformatic analysis of peroxidase genes in the soil bacterium *R*. *jostii* RHA1 genome sequence identified two DyP genes *DypA* and *DypB* ^45^. A study of gene deletion mutants using a colorimetric assay showed greatly reduced lignin degradation activity for the *∆dypB* mutant revealing its role in lignin degradation, while the recombinant DypB catalyzes oxidative Cα–Cβ cleavage of a β-aryl ether lignin model compound, and Mn^II^ to Mn^III[45]^. Given that the DypB is the first bacterial enzyme to be well-characterized for oxidation of polymeric lignin in wheat straw as well as hardwood Kraft lignin^38,48,51^, we were interested to heterologously express this protein *in planta* to see whether it maintains its catalytic activity to depolymerize lignin. Moreover, since targeting of proteins to the Endoplasmic Reticulum (ER) has been shown to improve protein accumulation, folding, stability and reduce protein degradation^52,53^, and to sequester the protein away from the cell wall where lignin polymerization takes place, we were also interested to target the protein to the ER.

Here we report that heterologous expression of the *R*. *jostii* RHA1 DypB in *N*. *benthamiana* and activation of the recombinant enzyme *in situ* improved subsequent saccharification by a cocktail of cellulase and glucosidase enzymes releasing remarkably more fermentable sugars, likely due to lignin depolymerization *in planta*. Moreover, targeting the protein to the ER enhanced protein accumulation without interfering with plant growth and development, revealing the potential of such transgenic plants as biofactories for large-scale production of ligninolytic enzymes. To our knowledge, this is the first attempt to express a lignin-degrading enzyme *in planta* that leads to improved lignocellulosic biomass saccharification.

## Results

### Characterization of DypB expressing transgenic tobacco

**Figure 1:**
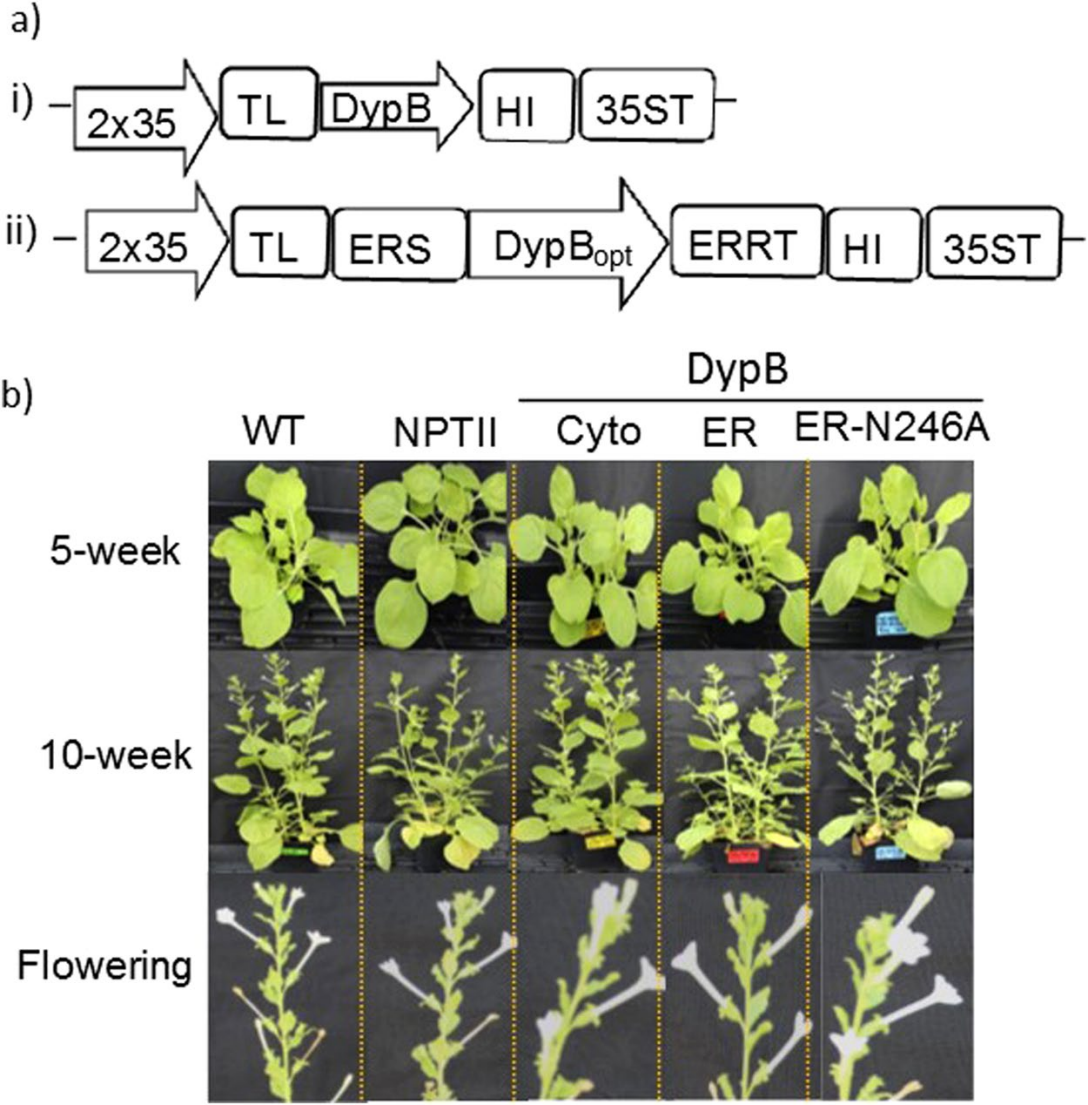
Heterologous expression of DypB in tobacco. (**a**) (i) Constructs of DypB for cytosolic (pPZP-NPTII-DypB) and (ii) ER-targeting pPZP-NPTII-DypB_opt_ under the control of enhanced 35S CaMV promoter (35S) and tobacco etch virus leader sequence (TL). For ER-targeting, the sequence was optimized to tobacco-preferred codons and flanked by an N-terminal ER signal peptide (ERSP) and a retention signal (ERRT). A polyhistidine tag (HIS) was fused to the sequences at the C-terminus to enable protein purification. (**b**) Phenotypes of transgenic and non-transformed tobacco lines at different developmental stages. The N246A lines express a variant of ER construct as in (ii) with Asn246 substitution by an alanine.

In this study, we generated expression constructs of the DypB with the native sequence for cytosol- (pPZP-NPTII-DypB, designated as Cyto) or sequence optimized for endoplasmic reticulum-targeting (pPZP-NPTII-DypB-DypB_opt,_ designated as ER, or ER-N246A, which is the variant with a single amino acid substitution that has been shown to increase catalytic efficiency^54^ (Fig. 1a). These constructs and the empty vector control (pPZP-NPTII) were introduced into *N*. *benthamiana*. At least ten T_0_ transgenic lines were generated for each construct, and seeds of the T_0_ lines were germinated to obtain T_1_ plants. Five independent T_1_ lines were characterized in detail for each construct. Integration of transgene into the genome of tobacco was confirmed by PCR amplification from genomic DNA isolated from at least four independent lines (Supplementary Fig. S1b).

Transgenic lines in which the DypB was expressed in the cytosol (Cyto, Fig. 1a (i)) or ER (ER, Fig. 1a (ii)) were phenotypically not different from the wild type and the empty vector control (Fig. 1c). Vegetative growth, flowering characteristics, and seed setting did not vary markedly between the transgenic lines and the control lines. This suggests that targeted accumulation of the DypB protein in the subcellular compartments (cytosol or ER) does not interfere with normal plant growth and development.

Although we did not observe marked differences between the tobacco lines during most of the developmental stages, the growth of one of the cytosol-targeting lines (Cyto7), at seedling stage on ½ MS media containing 50 mg/L Kanamycin was slower than the other transgenic lines (Supplementary Fig. S1). This may be due to lower activity of the aminoglycoside 3′-phosphotransferase (*aph* (3′)-II or NPTII) enzyme, which inactivates aminoglycoside antibiotics such as kanamycin by phosphorylation^55^. In the control without Kanamycin, however, the growth of Cyto7 was not different from the wild type and the other DypB-expressing lines. Moreover, after the seedlings were transferred to soil, the discrepancy in the rate of growth between Cyto7 and other lines disappears.

### Intracellular localization and DypB expression in transgenic tobacco

Intracellular targeting of the DypB constructs was studied by transient expression of GFP-fusion protein in tobacco epidermal cells. Images of tobacco leaves infiltrated with Agrobacterium harboring the enhanced GFP-fusion constructs (GFP::DypB_Cyto_ and GFP::DypB_ER_) were taken four days after infiltration. As shown in Supplementary S2, GFP signals were detected in leaves infiltrated with the control and fusion constructs. Signal of control GFP was detected in the majority of the cells and diffused in the cytoplasm (Supplementary Fig. S2a). Likewise, the signal of GFP::DypB_Cyto_ was detected in the cytoplasm surrounding the vacuole (Supplementary Fig. S2b), although there were fewer fluorescing cells as compared to the control and ER-constructs. In contrast, the signal of the ER-targeting GFP::DypB_ER_ appears to localize to the ER lumen forming an extensive network (Supplementary Fig. S2c). However, over time the signal appeared to spread to other cellular compartments which could be due to overexpression by the enhanced CaMV 35s promoter and saturation of the signal as previously reported^56^. The pattern of localization and signal intensity of GFP::DypB_ER_ is similar to that of the control ER-marker^57^ (Supplementary Fig. S2d), which confirms trafficking of the proteins to the target organelle.

**Figure 2:**
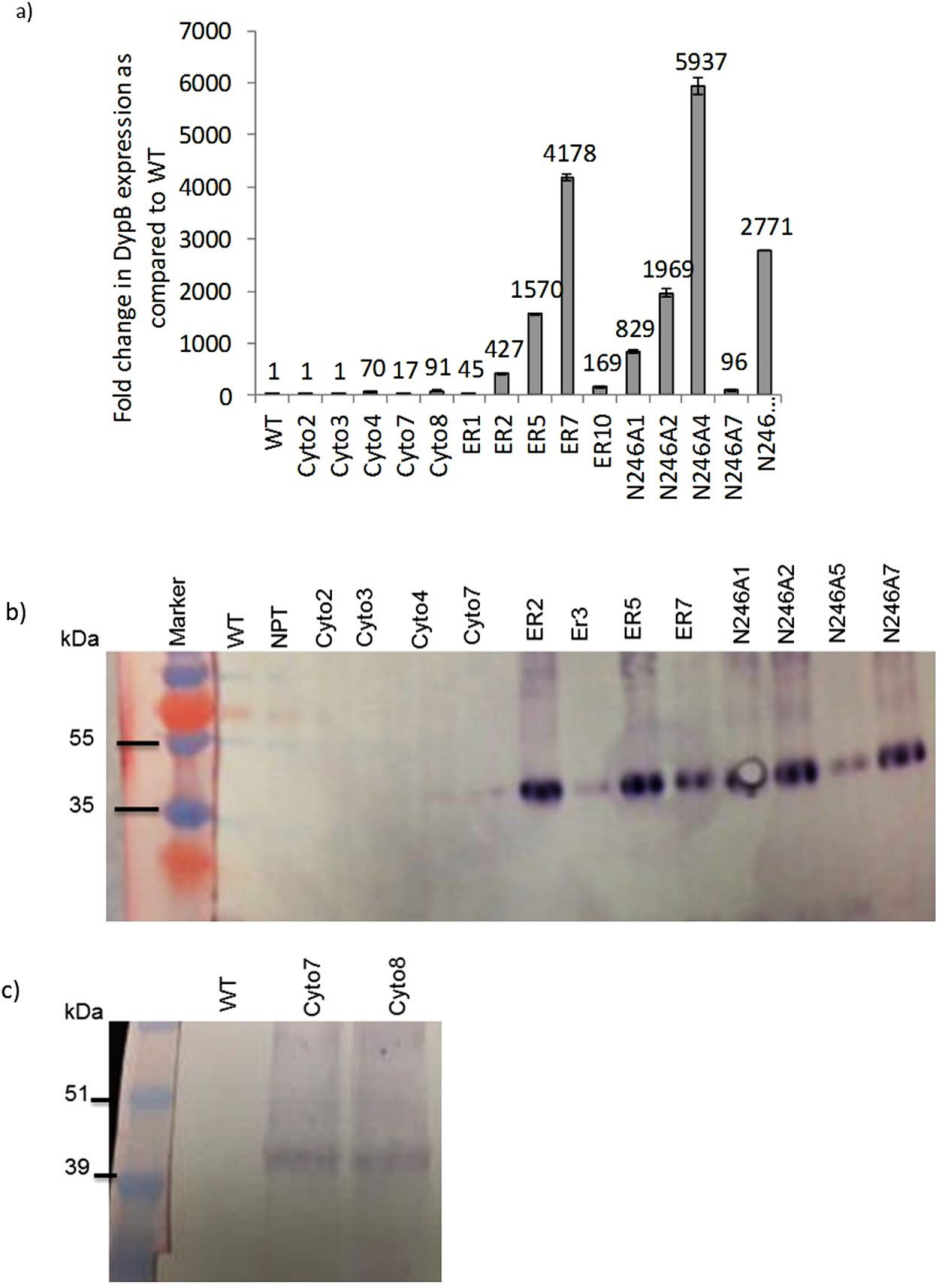
Analysis of DypB expression in transgenic *N*. *benthamiana*. (**a**) Quantitative real-time PCR. First-strand cDNA was synthesized from total RNA isolated from two-month-old greenhouse established plants as described in the ‘Materials and Methods’. Transcript accumulation in the WT sample was used as a reference to determine the ‘Fold Change’ in expression. (**b**) Western blot using anti HIS antibody. (**c**) Western blot using anti-DypB antibody. Total leaf soluble protein was extracted and purified from at least four independent transgenic lines per construct. The protein samples were separated on SDS-PAGE along with the WT and NPTII controls. The DypB was detected using anti-His polyclonal (**b**) and anti-DypB antibodies (**c**) as described in the ‘Materials and Methods’ section.

Expression of the DypB gene was studied using both quantitative real-time PCR and antibody-based protein detection by Western blotting. As shown in Fig. 2a, a remarkable increase in transcripts of DypB was observed for most of the transgenic lines. This may be attributed to the enhanced 35S promoter that enables generation of high copies of messenger RNA (mRNA), and position of the transgene insertion into the genome of tobacco. There was no increase in transcript level in two DypB_cyto_ lines (Cyto2 and Cyto3) while transcript level increased by 70, 17 and 90-fold for lines Cyto4, Cyto7 and Cyto8, respectively (Fig. 2a). Transcript levels were much higher for ER-targeting constructs, 45 to over 4000-fold for DypB_opt_ER, and 96 to nearly 6000-fold for N246A, suggesting a higher level of the bacterial DypB transcript accumulation in tobacco. Expression at the protein level was determined by using anti-His antibody quantification from purified leaf proteins. Consistent with the transcript data, stronger band intensity (~40 kDa) for the DypB protein was detected for ER targeting (ER or N246A) constructs (Fig. 2b). DypB product in the cytosol was weakest (Fig. 2c) with the protein only detected after increasing the loading rate by 10-fold (DypB_cyto_), suggesting a low level of DypB protein accumulation in the cytosol. This could be due to lower level of transcription and subsequence translation or a higher rate of protein degradation in the cytosol. As expected, the DypB protein was not detected in the wild type and empty-vector control (NPTII) lines (Fig. 2b and c).

### Enzymatic activity of purified DypB

**Figure 3:**
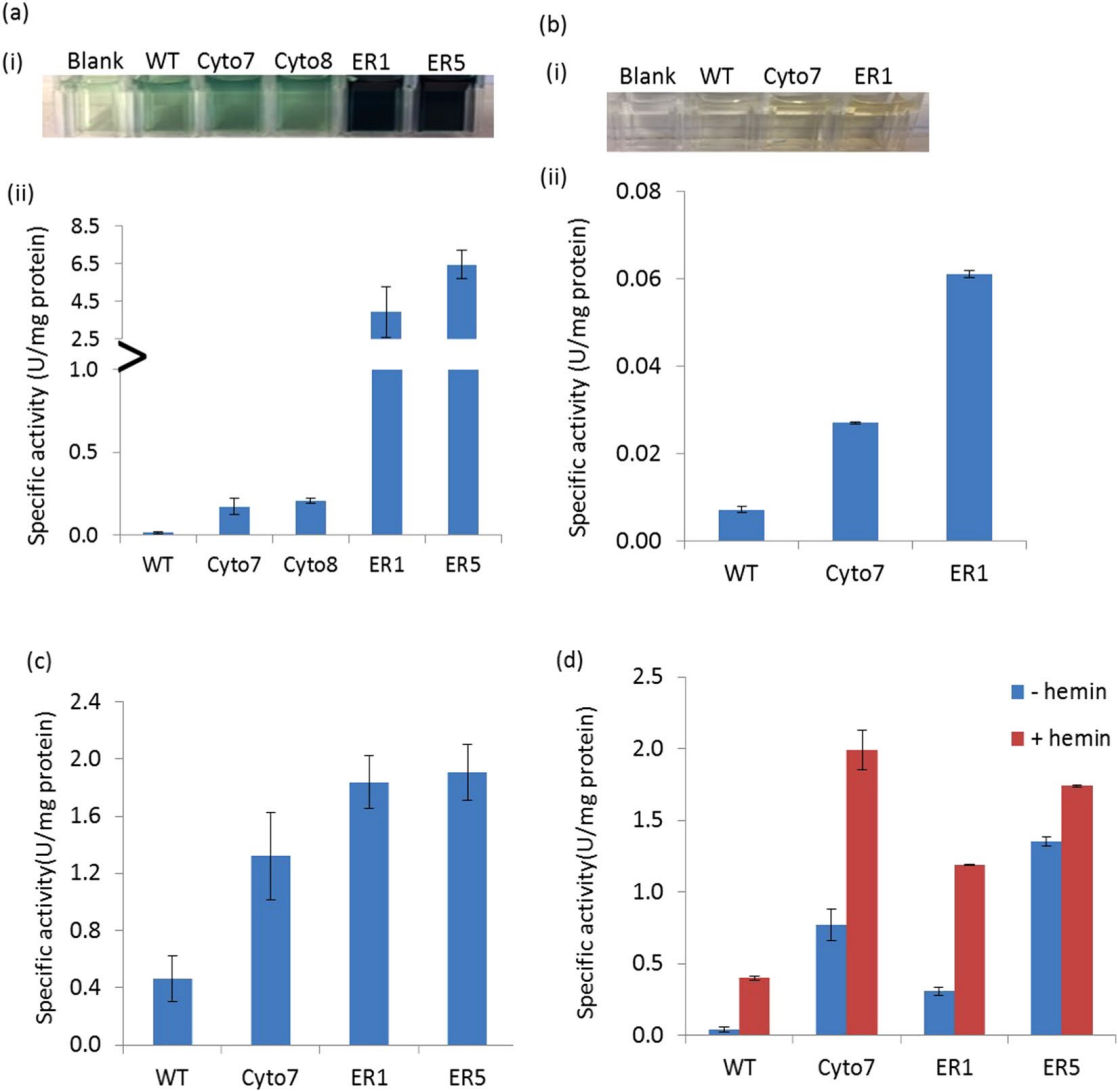
Activity of DypB purified from transgenic *N*. *benthamiana* on 10 mM ABTS (**a**), 1 mM DMP (**b**), Kraft lignin (**c**) and VA (**d**). Assay was performed in 50 mM Na-acetate (ABTS and DMP) and succinate buffer (Kraft lignin) (pH 5.5), 110 mM sodium tartrate (VA) buffer pH 3.0, in the presence of 2.5 mM MnCl_2_ (ABTS, DMP and VA) and 10 mM MnCl_2_ (Kraft lignin), 1 mM H_2_O_2_ and 50 μL of purified protein. The reactions were initiated by adding the H_2_O_2_ and increase in absorbance was measured after 1 min at 420, 468, 465 and 310 nm for ABTS, DMP, Kraft lignin and VA, respectively. Bars in (**a**, ii), (**b**, ii), (**c**) and (**d**) represent enzyme specific activity in micromoles of each substrates oxidized per minute per milligram protein from WT and transgenic lines.

Since peroxidases including the DyPs have been implicated in the oxidation of model and lignin-related compounds including 2, 6-dimethylphenol (DMP), 2, 2′-azino-bis (3-ethylbenzothiazoline-6-sulphonic acid (ABTS), Kraft lignin and veratryl alcohol (VA)^45,49,58^, we tested the specific activity of the recombinant DypB purified from representative lines on these compounds. As shown in Fig. 3a, the level of ABTS oxidation was considerably higher for enzyme extract from all the tested transgenic lines as compared to the wild type control (WT). The highest activity was observed for the ER-targeting lines ER5 followed by ER1, and the cytosolic-targeting lines Cyto7 and Cyto8. Similarly, extracts from Cyto7 and ER1 showed higher activity in degrading the phenolic compound DMP than the wild type control (Fig. 3b). Similarly, the enzyme extracts from the transgenic lines showed higher activity in degrading lignin-related substrates Kraft lignin (Fig. 3c) and VA (Fig. 3d). Higher activity of Kraft lignin oxidation (four-fold) was observed for enzymes of the ER-targeting lines than WT control. The activity of the Cyto7 enzyme was also about three-fold higher than the WT. As shown in Fig. 3d, the activity of Cyto7 and ER5 in degrading VA is 20-and 30-fold higher than the WT, respectively. Interestingly, this activity was enhanced in the presence of hemin. However, since the recombinant DypB was active in the absence of hemin, extraction and purification procedures do not seem to markedly affect protein structure and catalytic properties. Moreover, for the reactions with Kraft lignin and VA, maximum absorbance values were measured in less than two minutes, and the values did not markedly increase afterwards. Future research should include a detailed kinetic study to determine whether this is due to high affinity of the recombinant DypB to these substrates. Overall, these findings suggest that the bacterial peroxidase is expressed in an active form in tobacco, and subcellular compartmentalization of the enzyme enhanced protein expression without modulating its catalytic properties.

### Enzymatic saccharification of control and transgenic tobacco biomass

Given that the recombinant DypB enzyme extracted from transgenic tobacco showed higher activity in oxidizing model and lignin-related compounds *in vitro* as compared to extracts from non-transformed control plants (ref.^45^ Fig. 3), we were interested to test whether the enzyme could be active *in planta* prior to biomass saccharification and indeed, if active, could result in increased sugar release. Biomass was incubated in DypB activation buffer containing MnCl_2_ and H_2_O_2_ for three days, and then subjected to hydrolysis by a mixture of cellulase and glucosidase enzymes for three more days before the hydrolysate was used for sugar analysis. The concentration of major hexoses (glucose, galactose and mannose) and pentoses (xylose and arabinose) from filtered hydrolysate was determined after calibration with standards of known sugar concentration.

**Figure 4:**
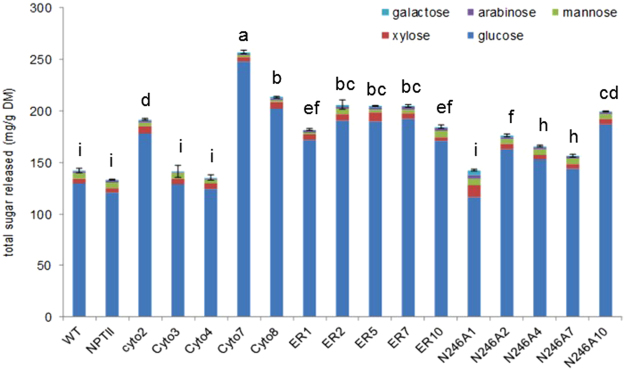
Release of hexose and pentose sugars from non-transformed (WT) and empty vector (NPTII) controls, and representative transgenic lines. The DypB was activated directly in the biomass as described in the enzyme activity assay method prior to saccharification by a cocktail of cellulase and glucosidase. Glucose yield was determined in the hydrolysate from at least four replicates using IC-DIONEX (different letters indicate statistically significant differences (p < 0.0001).

As presented in Fig. 4 and Supplementary Table S1, both hexose and pentose sugars were released after biomass saccharification. Marked differences were observed in the amount of sugars released from the tobacco lines. The majority of the transgenic lines released significantly more glucose than the non-transformed wild type (p <0.0001), and the empty vector control (NPTII) lines. Glucose was the dominant sugar released from all the tobacco lines (transgenic as well as control lines). Interestingly, cytosolic-targeting lines (Cyto7 and Cyto8), released the highest amount of glucose; accounting for up to 96% of the total sugars. The amount of glucose released from two cytoplasmic-targeting lines (Cyto3 and Cyto4) did not differ from the non-transformed control while three lines (Cyto5, Cytro7 and Cyto8) released 38–91% more glucose than the non-transgenic control plants. The ER-targeting lines released 32–49% more glucose than the non-transgenic line (Fig. 4 and Supplementary Table S1). Similarly, four lines of N246A (N246A2, N246A5, N246A7, N246A10), which is a single amino acid substitution to the ER construct, released 11–44% more glucose than the control plants while N246A1 released only 90% of that of the wild type (Fig. 4 and Supplementary Table S1). Interestingly, N246A1 released substantially more pentose sugars (xylose and arabinose), which are derived from hemicellulose^59^, than the other transgenic lines or the control. However, even for N246A1 the pentose sugar release per gram dry matter (16 mg g^−1^ DM) was an order of magnitude lower than the glucose released across all the tobacco lines (120–250 mg g^−1^ DM) (Fig. 4, Supplementary Table S1). The amount of galactose released from the transgenic lines, except Cyto3 and Cyto4, was higher than the control lines. However, only three transgenic lines Cyto2, ER2 and ER5 released more xylose than the wild type. Lines ER7, ER10 and N246A1 released more arabinose than that of the wild type control. On the other hand, none of the transgenic lines released significantly more mannose than the control (Fig. 4 and Supplementary Table S1) while the amount released from Cyto2, Cyto4, Cyto7, Cyto8, ER1 and ER5 was even lower than that of the control. Taken together, these findings indicate that heterologous expression of DypB improves biomass saccharification efficiency. This observation is consistent with activation of DypB *in planta*, leading to lignin degradation and ultimately improving accessibility of cell wall for saccharification by a cocktail of cellulase and glucosidase enzymes.

### Biomass pyrolysis and GC-MS analysis

Our findings reveal that most of the transgenic tobacco lines released significantly more fermentable sugars than the non-transformed control. Given that the DypB was compartmentalized in the cytosol and ER, *in situ* modification of lignin in the cell wall following polymerization can be ruled out. Therefore, to decipher the underlying biochemical mechanisms for the improved biomass digestibility, we performed pyrolysis*-*GC/MS analysis before or after activation of the DypB enzyme directly in the biomass to see whether the observed increase in saccharification efficiency in the transgenic lines was achieved by lignin modification *in planta*. Shoot biomass including stem, branches, leaves, inflorescence and seeds obtained from the upper 25% of the shoot was ground, vacuum dried, and treated for DypB activation as described in the ‘Materials and Methods’ section or dried and directly used for pyrolysis.

The analysis showed an overall decrease in the abundance of pyrolysates associated with lignin in the transgenic lines as compared to the wild type (Table 1, Supplemental Fig. 3). Moreover, DypB-activated samples in each line demonstrated a decrease in the overall lignin-associated pyrolysates compared with their non-activated counterparts. Pyrolysates reported to be derived from lignin^8,9,60,61^, including phenol, 2-methyl-phenol, 4-methyl-phenol, 2, 4-dimethoxy-phenol and hydroquinone, showed a marked decrease in the transgenic lines than was observed in non-transformed and empty-vector control lines, most noticeably from samples in which the enzyme was activated prior to pyrolysis. This line of evidence suggests the activated enzymes accelerated degradation of lignin. However, we cannot rule out that various other factors contributed to the decrease in the abundance of these pyrolysates, including overall decrease in the abundance of lignin polymers, and differing ratios of plant parts (stem nodes, internodes, leaves and seeds) among the transgenic lines as compared to the wild type control. As reported previously from a study involving sorghum^7^, tissues taken from different parts of the plant can differ widely in the type and amount of lignin present. Therefore, any change in the weight ratio of the various plant parts in the analyzed samples could result in a shift in the lignin content.

**Table 1:** Comparison of pyrolysates (% area of program) from control and transgenic tobacco biomass. Wild type and transgenic tobacco biomass was pyrolyzed at 650 °C with (treated) and without (untreated) activating the recombinant DypB in the biomass. Mean values represent percent area of the pyrograms and standard errors (n = 2).

Compounds	Wild type		NPTII		Cyto		ER		N246A	
	Untreated	Treated	Untreated	Treated	Untreated	Treated	Untreated	Treated	Untreated	Treated
Acetic acid	11.49+3.19	12.16+6.11	11.25+0.86	12.36+1.14	10.62+1.48	9.85 ± 1.36	11.89 ± 0.90	11.85 ± 1.25	10.32 ± 1.97	10.17 ± 0.66
Toluene	1.08 ± 0.51	1.09 ± 0.63	0.93 ± 0.22	0.72 ± 0.63	0.38 ± 0.17	0.23 ± 0.18	0.87 ± 0.21	0.36 ± 0.25	0.54 ± 0.24	0.27 ± 3.19
Furfural	2.03 ± 0.61	3.45 ± 0.70	1.47 ± 0.56	2.72 ± 0.48	1.60 ± 0.22	2.02 ± 0.31	1.41 ± 0.22	2.31 ± 0.23	1.50 ± 0.29	1.86 ± 0.66
Phenol	1.70 ± 0.18	0.97 ± 0.29	1.62 ± 0.13	0.85 ± 0.47	1.108 ± 0.20	0.33 ± 0.15	1.86 ± 0.34	0.40 ± 0.18	1.07 ± 0.15	0.39 ± 0.10
Phenol, 2-methoxy-	1.58 ± 0.31	1.05 ± 0.29	1.39 ± 0.16	0.94 ± 0.16	1.14 ± 0.22	0.57 ± 0.26	1.58 ± 0.17	0.83 ± 0.10	1.29 ± 0.16	0.77 ± 0.08
Phenol, 2-methyl-	0.80 ± 0.37	0.49 ± 0.28	0.71 ± 0.10	0.32 ± 0.27	0.29 ± 0.14	0.05 ± 0.10	0.58 ± 0.28	0.00 ± 0.00	0.30 ± 0.22	0.00 ± 0.00
Phenol, 4-methyl-	0.47 ± 0.22	0.52 ± 0.31	0.54 ± 0.04	0.35 ± 0.19	0.21 ± 0.17	0.13 ± 0.09	0.50 ± 0.23	0.13 ± 0.12	0.21 ± 0.17	0.12 ± 0.09
Phenol, 2,4-dimethyl-	0.45 ± 0.26	0.09 ± 0.09	0.41 ± 0.05	0.16 ± 0.16	0.11 ± 0.12	0.00 ± 0.00	0.29 ± 0.21	0.00 ± 0.00	0.15 ± 0.15	0.00 ± 0.00
Phenol, 2-methoxy-4-methyl-	0.00 ± 0.00	1.01 ± 0.77	0.11 ± 0.24	0.87 ± 0.67	0.00 ± 0.00	0.48 ± 0.46	0.00 ± 0.00	0.98 ± 0.73	0.00 ± 0.00	1.32 ± 0.27
2-Methoxy-4-Vinylphenol	1.13 ± 0.32	0.95 ± 0.21	1.08 ± 0.22	0.85 ± 0.21	0.98 ± 0.16	0.75 ± 0.15	1.08 ± 0.12	0.85 ± 0.10	1.07 ± 0.08	0.88 ± 0.19
Phenol, 2,6-dimethoxy-	2.51 ± 0.85	1.65 ± 0.71	2.29 ± 0.51	0.92 ± 0.27	1.85 ± 0.43	1.02 ± 0.34	2.14 ± 0.51	1.24 ± 0.63	1.95 ± 0.28	0.88 ± 0.26
Phenol, 2-methoxy-4-(1-propenyl)-, (E)-	0.84 ± 0.45	0.96 ± 0.27	0.79 ± 0.09	0.85 ± 0.07	0.65 ± 0.07	0.63 ± 0.11	0.65 ± 0.30	0.77 ± 0.08	0.70 ± 0.09	0.78 ± 0.14
Hydroquinone	1.30 ± 0.61	0.26 ± 0.31	2.17 ± 0.31	0.48 ± 0.41	1.21 ± 0.33	0.00 ± 0.00	2.26 ± 0.56	0.00 ± 0.00	1.38 ± 0.22	0.00 ± 0.00
Phenol, 2,6-dimethoxy-4-(2-propenyl)-	0.70 ± 0.71	0.78 ± 0.43	0.56 ± 0.04	0.96 ± 0.46	0.96 ± 0.29	0.79 ± 0.78	1.03 ± 0.24	0.55 ± 0.14	0.74 ± 0.28	0.64 ± 0.54
D-Allose	0.00 ± 0.00	3.33 ± 2.07	0.16 ± 0.23	6.66 ± 3.32	0.60 ± 0.85	1.50 ± 1.15	0.00 ± 0.00	10.83 ± 2.56	0.33 ± 0.46	8.16 ± 4.05
n-Hexadecanoic acid	3.82 ± 0.62	3.42 ± 2.00	4.82 ± 1.08	7.70 ± 0.91	6.22 ± 1.02	5.00 ± 1.13	5.12 ± 0.53	7.05 ± 2.95	7.08 ± 1.34	8.02 ± 1.51
9,12-Octadecadienoic acid (Z,Z)-	8.26 + 2.48	1.87 + 2.08	13.48 + 1.46	1.96 + 1.32	24.03 + 7.10	7.88 + 6.09	21.29 + 2.95	2.43 + 0.94	24.05 + 2.54	23.35 + 2.04

The NPTII control lines showed no marked difference from the wild type in the amount of pyrolysate except for the presence of higher lipid content (n-Hexadecanoic acid and 9, 12-octadecadienoic acid (Z, Z)), which was even more pronounced in the transgenic lines (2-3 times higher than the wild type). The level of lipid content is higher than the typical levels observed after biomass pyrolysis, which appears to be due to inclusion of seeds in the samples, leading to a decrease in lignin content since these values are based on percent of peak areas. Although we have not precisely determined the seed yield in each tobacco line and the amount of seeds in each pyrolyzed sample, we have not observed a marked difference in seed production among the lines, and also we maintained uniformity of tissue prior to grinding for pyrolysis. Hence, the observed decrease in the amount of lignin-associated pyrolysate between the transgenic and control plants is likely a consequence of *in planta* lignin degradation due to DypB activation prior to pyrolysis, resulting in the release of lignin monomers into the activation buffer.

Similar to the lignin-associated pyrolysates which showed a decrease in the activated samples, the lipid (9, 12-octadecadienoic acid (Z, Z)) content also decreases substantially in all treated samples except for the N246A lines. This too may be a result of several factors. For example, manganese absorbed by the biomass may be catalyzing side reactions that lead to additional lignin charring, which is a chemical process of incomplete combustion when the plant tissue is subjected to a high heat, facilitating the liberation of carbohydrates. To overcome this problem, the plant tissue was rinsed with 50 mL of deionized (DI) water after activation; however, a thorough effort to remove manganese may be necessary. Furthermore, analyzing the different plant tissues separately would minimize variability in the analysis which will be addressed in subsequent studies.

### Biomass composition analysis

**Figure 5:**
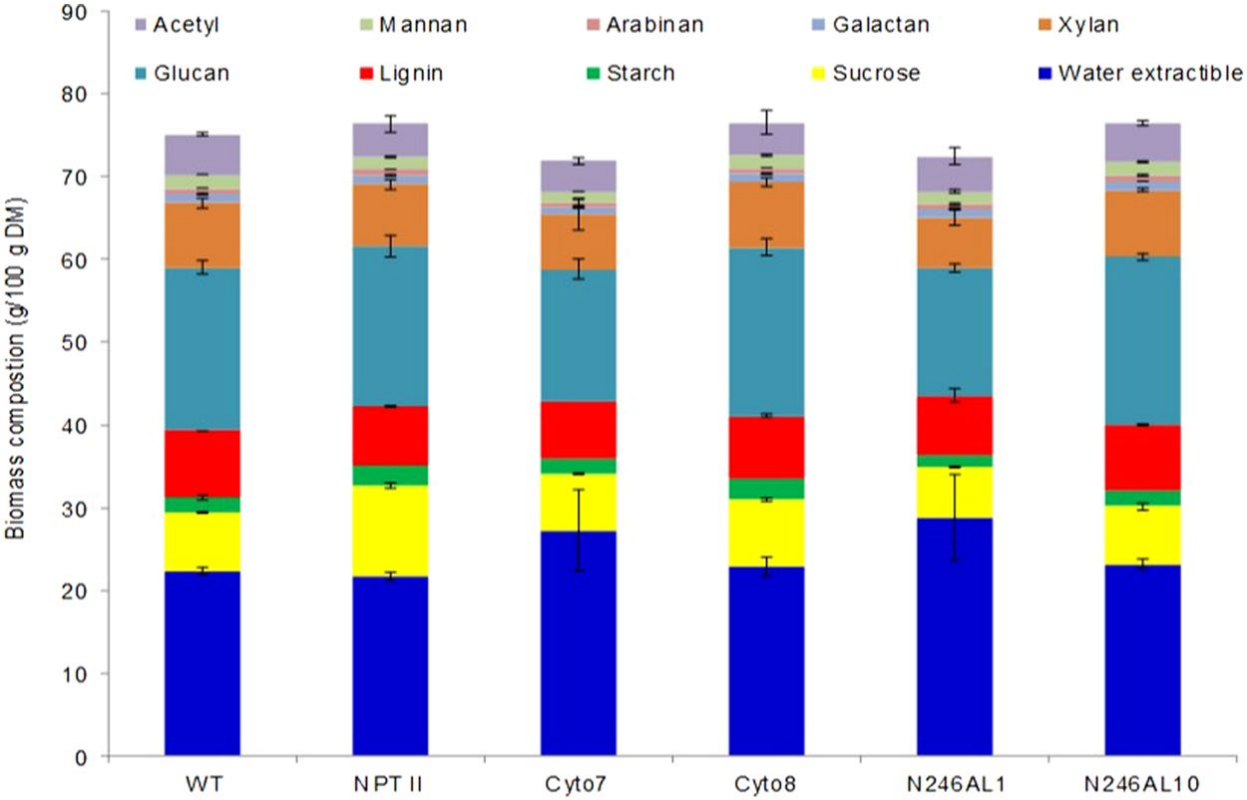
Composition analysis of tobacco biomass according to NREL procedures. Structural and non-structural carbohydrates, acetyl and lignin were quantified in non-transformed (WT) and empty vector (NPTII) controls and representative transgenic lines (Cyto7, Cyto8, N246A1 and N246A10).

To understand whether variation in the initial biomass composition could have accounted for the observed differences in sugar release among the tobacco lines, biomass composition was analyzed according to NREL^62,63^, and Van Soest^64^ procedures. Interestingly, while the absolute results differed as a result of the protocols, both procedures did not detect a marked variation among the various transgenic lines and controls for both structural and non-structural carbohydrates as well as lignin (Fig. 5, Supplementary Tables S2 and S3). Based on the NREL method, lignin content varied from 7.0% in Cyto7 to 8.1% in the WT control **(**Fig. 5 and Supplementary Table S2 **)** while glucan content varied from 15.5% in N246A1 to 20.4% in Cyto8, and for the Cyto7 line that released the highest amount of glucose, the glucan content was 16.0% as compared to WT (19.7%). On the other hand the percent of water extractible components was slightly higher in N246A1 (28.8%) and Cyto7 (27.2%) than WT (22.4%). Analysis using the Van Soest method detected lower lignin content (3.3 to 4.2%) than the NREL method. which could be due to loss of lignin during sample preparation. There was also a mild variation in cellulose and hemicellulose content across the different transgenic lines (Supplementary Table S3 **)**. However, the observed modest variation in the content of structural and non-structural carbohydrates and lignin does not appear to explain the remarkable increase in the release of sugars from the transgenic lines as compared to the non-transgenic control (Fig. 5 and Supplementary Table S1).

## Discussion

The genome of *R*. *jostii* RHA1 contains two genes (DypA and DypB) encoding members of the DyP superfamily^45^. Both proteins exhibit peroxidase activity using a chromogenic compound ABTS, pyrogallol and RB4 in H_2_O_2_-dependent manner, but only DypB is implicated in oxidizing Mn^II [44,54]^ and polymeric lignin also in a H_2_O_2_-dependent manner^45^.

### Phenotype of DypB expressing transgenic plants

In this study, the DypB was expressed in tobacco without a signal peptide for cytosol-targeting or with an N-terminus signal peptide and a C-terminus retention signal for ER-targeting. Importantly, the transgenic lines were not phenotypically different from the non-transformed control (Fig. 1), suggesting that the DypB did not interfere with lignin deposition and normal plant growth and development despite its ability to degrade model lignin compounds as well as lignocelluloses^53,65^. We hypothesize this is due to sequestration of the enzyme in the intracellular compartments away from the apoplast, where lignin polymerization takes place. Targeting the DypB to the apoplast may lead to *in situ* lignin degradation which may interfere with plant fitness since lignin plays an important role, for example, in providing structural support, water transport and protection against chemical and biological attack^10,66,67^. Reduced plant lignin content caused by mutation, trait improvement though breeding or transgenic approaches may negatively impact their agricultural fitness, while positive or absence of effect have also been reported^68,69^. Transgenic expression of other classes of fungal peroxidases such as the *P*. *chrysosporium* manganese peroxidase isozyme H3 (MnP-2) in tobacco chloroplast^70^, MnP from *Coriolus versicolor* ^71^ and the secretory *Trametes*. *versicolor* LiP^72^ also in tobacco, and *T*. *versicolor* MnP^73^ in hybrid aspen, did not markedly affect plant growth. In contrast, transgenic accumulation of MnP from *P*. *chrysosporium* in the ER of alfalfa^74^ and maize^75^ adversely affected plant growth and development depending on the level of protein accumulation. Transgenic alfalfa plants showed growth stunting and yellowing of foliage^74^ while maize plants expressing the MnP showed leaf lesions and were capable of producing seeds^73^, which may be due to metabolic burden of accumulation and maintenance of a foreign protein, for example, through competition for precursors that are otherwise utilized for maintaining normal plant growth.

### Accumulation of the DypB in the ER of transgenic tobacco

Heterologous protein accumulation in the intracellular compartments has been an effective strategy to increase protein yield and stability in plants^76–78^. Accordingly, a number of plant cell wall-modifying enzymes have been accumulated at higher levels in plant cellular compartments such as the ER, chloroplast, mitochondria or the apoplast^23,27,78,79^. In this study, targeting of DypB to the ER of tobacco under the control of enhanced 35S promoter and tobacco etch virus translation enhancer^80^ led to increased protein level as detected by anti-HIS antibody (Fig. 2). This is achieved likely due to both N-terminal signal peptide and the ER tetrapeptide retention signal (HDEL)^81^. Simultaneous fusion of the signal peptide and the retention motif to the N-and C-termini, respectively, of recombinant proteins enables retrieval of the proteins from the Golgi apparatus to the ER lumen, resulting in more than 10–100 fold enhancement as compared to without the retention motif^52,82,83^. Moreover, the *DypB* nucleotide sequence used for ER-targeting was codon-optimized for *N*. *benthamiana*, and the constructs have an N-terminus tobacco etch virus leader sequence which has been shown to enhance mRNA translation^80,83^. Furthermore, ER-targeting has a number of advantages including the presence of a suitable environment for correct folding and disulfide bridge formation by the ER-chaperones, such as binding proteins (BiP) and protein disulfide isomerase (PDI)^52,53^, and low levels of protolytic activity in the ER-lumen as compared to the cytosol. Various recombinant proteins including industrial enzymes, medical antibodies and antigens, and other therapeutic proteins have been successfully produced in plants^84,85^. Increased accumulation of the DypB in *N*. *benthamiana* in this study suggests that plants can be used as a biofactories for the production of ligninolytic enzymes.

In contrast with the ER targeted construct, the DypB protein was barely detected for the cytosolic-targeting construct (Fig. 2). This is likely due to either the native bacterial sequence not being optimized for tobacco translation machinery, resulting in low DypB protein synthesis, or due to a position effect (*i*. *e*. site of the transgene insertion on the chromosome)^86,87^ resulting in reduced transcription of the protein as compared to the ER-targeting lines. Generally, recombinant proteins targeted to the cytosol are detected at very low levels despite high mRNA levels, resulting in accumulation rates below 0.1% of total soluble protein (TSP) in several cases^88,89^. For example, cytosol targeting of the tomato mosaic virus antibody ‘rAb29’^60^ and a human growth hormone^90^ in tobacco leaf resulted in very weak accumulation rates (0.01–0.1% of TSP). Interestingly, the same transgenes coupled with a signal peptide for extracellular secretion^52^ or apoplast^90^ resulted in accumulations up to 10% of TSP. Low levels of cytosol expression could also be due to unfavorable redox potential^91^ as well as important post-translational modifications (such as glycosylation), which may modulate folding, assembly and/or structural stability of several proteins^92^, and/or the effective housekeeping activity of the ubiquitin–proteasome proteolytic pathway^93,94^, which is involved in the recognition and degradation of incorrectly folded proteins. However, stable and high-level expression of some recombinant proteins in the cytosol has also been reported^95^.

### Catalytic activity of recombinant DypB *in vitro*

Previous characterization of the DypB catalytic properties revealed that it catalyzes the peroxide-dependent oxidation of lignin and divalent manganese (Mn^2+^)^45,51,64^. In this study recombinant DypB purified from the leaves of transgenic lines consistently showed higher peroxidase activity than extract from the wild type control in oxidizing standard peroxidase substrates ABTS and DMP as well as lignin-related substrates Kraft lignin and VA (Fig. 3), indicating that functionally active DypB protein can be produced *in planta*. Generally, the specificity constant (*k*_*cat*_/*K*_*m*_) values for A and B-type Dyps for ABTS is in three orders of magnitude lower than those of C- and D-type DyPs^46,48,50^. The Dyp1B enzyme recently isolated from *Pseudomonas fluorescens* Pf-5 has been shown to oxidize Kraft lignin and Mn^2+^, releasing an oxidized lignin dimer in the presence of Mn^2+^ ^96^. Likewise, a DyP-type C peroxidase enzyme that has been identified from the soil bacterium *Amycolatopsis sp*. 75iv2 ATCC 39116, shows Mn^2+^ oxidation activity with much higher catalytic efficiency than *R*. *jostii* RHA1 DypB, approaching the activity of fungal Mn peroxidase enzymes^45^. An effort towards improving the specific activity of the DypB through substitution of an active site Asn246 in DypB by Ala has been found to increase the k_cat_ for Mn^2+^ oxidation 80-fold^54^, suggesting a potential for improving the activity of these enzymes through protein engineering, for example, via substitution of amino acid residues and/motifs by directed evolution or saturated mutagenesis^97,98^ or domain-swapping with more active DyPs such as the C- or D-type subfamily.

### *In situ* activation of the DypB improves biomass saccharification

Although plant heterologous expression of ligninolytic enzymes such as LiP and MnP have been reported previously, those prior studies were aimed at studying the feasibility of recombinant enzyme production^70,71,99^ and phytoremediation of toxic chemicals from the environment^71–73^. Accumulation of lignin degrading enzymes such as LiP, MnP, DyPs and laccases with the purpose of improving saccharification efficiency has never been reported. Therefore, given that the DypB has been shown to degrade standard peroxidase substrates such as ABTS and DMP, lignin related compounds Kraft lignin and VA (Fig. 3) as well as lignocellulose *in vitro* [ref.^45^ and this study], we hypothesized that the DypB would be able to degrade intact lignin in the cell wall, and thus reduce biomass recalcitrance. While plant lignin content and structure varies with factors such as growth stage, genotype, morphological fraction (leaf blade, leaf petiole, stem, inflorescence), and environmental conditions^67^, tobacco (*N*. *benthamiana*) contains about 13% lignin^6^. In this study, the top portion of wild type and transgenic tobacco biomass was pulverized by mortar and pestle under liquid N_2_ and incubated in the presence of Mn^2+^ and H_2_O_2_ to activate the recombinant DypB *in planta*. The resulting biomass was subjected to saccharification by a cocktail of cellulase and glucosidase enzymes, prior to analysis of sugar species and yield in the hydrolysate.

The amount of fermentable sugars released from most of the transgenic lines was significantly higher than that of the non-transgenic control (Fig. 4). Glucose was the dominant sugar species released, accounting for over 80% of the total sugar in both control and transgenic lines. However, despite lower level of protein expression (Fig. 2b and c), and relatively lower enzymatic activity in Cyto7 as compared to ER lines (Fig. 3), the amount of glucose released from Cyto7 was higher than the ER lines. This discrepancy could be due to the biomass preparation method, which might have resulted in the release of more enzyme from Cyto7 and allowed interaction with lignin in the cell wall as compared to the ER lines, where the DypB may be more strongly sequestered in the ER organelle. Moreover, enzyme activity is also modulated by posttranslational modification such as protein phosphorylation, glycosylation, which might be more pronounced in the Cyto7 lines. However, further study is needed to determine the precise mechanism of regulation of the recombinant DypB, as well as how to maximize its efficacy.

This large increase in saccharification efficiency in the transgenic lines is likely due to depolymerization of intact lignin since there was no marked difference in the content of structural and non-structural carbohydrates and lignin (Fig. 5, and Supplementary Tables S2 and S3). Therefore, to decipher the underlying biochemical mechanisms for the improved cell wall digestibility, we performed pyrolysis*-GC-MS* analysis of biomass with or without preactivation of the DypB enzyme *in planta*. As presented in Table 1, acetic acid was the dominant compound of the pyrolysates in all the tobacco lines. Importantly, the amount of lignin degradation products such as phenol, 2-methyl-phenol, 4-methyl-phenol, 2, 4-dimethoxy-phenol and hydroquinone was lower in the transgenic lines than was observed in non-transformed and empty-vector control lines, most noticeably from samples in which the enzyme was activated prior to pyrolysis (Table 1). Given that the DypB protein was sequestered in the cytosol and the ER, *in situ* modification of intact lignin during polymerization can be ruled out. Therefore, the observed reduction in the amount of lignin associated pyrolysate is likely due to activation of the DypB enzyme *in situ*, which appears to have depolymerized lignin in the cell wall, releasing lignin monomers into the hydrolysate, and making cellulose accessible for saccharification. However, the decrease in pyrolysate yield may also be due to recondensation of lignin monomers because of DypB activation, and increased char formation. A model summarizing intracellular DypB accumulation, biomass processing, enzyme activation and mode of action, and subsequent saccharification or pyrolysis of the biomass is presented in Supplemental Fig. 4 to enable readers understand the processes reported in the current study.

To see whether lignin-monomers are released during *in situ* activation of the enzyme, the hydrolysate was extracted with a mixture of dichloromethane and ethylacetate, and the organic fractions were immediately analyzed by GC-MS after being concentrated and filtered. However, we were not able to detect compounds that are typically derived from lignin. The compounds that were detected included 1-cyclohexyl ethanol, 2-propane-1-ol-3-phenyl, benzyl alcohol, phenyl ethyl alcohol, and other compounds, many of which contained aromatic rings, and appeared to be more abundant in the hydrolysate from the transgenic lines as compared to the non-transformed control. Our inability to detect typical lignin degradation products may be due to radical-based reactions occurring during the activation and extraction procedures; in the absence of a mechanism to remove lignin fragments that are likely susceptible to oxidation and repolymerization, these may produce heavier lignin-based products that are beyond the detection or identification limit of GC-MS^100–103^. If the latter is the case, addition of radical inhibitor such as boric acid as a capping agent during the activation process^102^, and perhaps butylated hydroxytoluene during the organic extraction could help in the identification of uncompromised lignin products. This technique will be implemented in future studies. Other methods for future studies of lignin modification include the NMR technique previously applied to tobacco plants in which lignification enzymes cinnamyl alcohol dehydrogenase and cinnamoyl-CoA reductase were down-regulated^104^, and non-catalytic Pyrolysis-GC-MS which can be used to characterize lignin by its H-, G- or S subunits^105^.

### Mechanism of lignin degradation by DypB

Lignin degrading enzymes identified to date exhibit diverse modes of action. Lignin peroxidases such as LiPs catalyze oxidative cleavage of C–C or ether (C–O–C) bonds in non-phenolic aromatic substrates of high redox potential, and MnPs oxidize Mn^2+^ to Mn^3+^, which facilitates the degradation of phenolic compounds or, in turn, oxidizes a second mediator to the breakdown non-phenolic compounds^9^. The MnP from *P*. *chrysosporium* has been reported to catalyze Cα-Cβ cleavages, Cα-oxidation and alkyl-aryl cleavages of phenol syringyl type β-1 lignin structure^106^. Likewise, DypB has been shown to catalyze Cα-Cβ oxidative cleavage of a β-aryl ether lignin model compound releasing vanillin as a product^54^, this oxidative cleavage was inhibited by addition of diaphorase (EC 1.8.1.4), an enzyme that catalyzes di- and tri-phosphopyridine nucleotides-dependent reduction of various dyes^107^, consistent with a radical mechanism for C-C bond cleavage. Ahmad *et al*.^45^ also reported that DypB shows activity toward guaiacol and vanillin, and suggested that the mechanism is likely via one-electron oxidation of the phenolic ring, followed by C-C bond cleavage.

Our suggestion of *in planta* lignin degradation is consistent with previous reports demonstrating *in vitro* degradation of lignocellulosic substrates such as wheat straw lignocellulose and Kraft lignin by recombinant DyP-type peroxidases including DypB^45,51,54^ *Pseudomonas fluorescens* Dyp1B^38,96^ and *Irpex lacteus* DyP^108^. Some DyPs have been shown to oxidize substrates that are too large to fit in the active site. For example, DypB showed saturation kinetics towards the large molecules of Kraft lignin^65^. The authors suggested a long-range electron transfer (LRET) between the surface of DypB enzyme involving several residues and the hydrophobic substrate through Tyr287 and Asp288, which forms a hydrogen bond with His226, which is the fifth ligand to the heme cofactor. The LRET-pathway has also been reported for *Aau*DyPI of *Auricularia auricula*-*judae* ^109,110^ and a lignin peroxidase (LiP) from the plant superfamily of peroxidases^111,112^, while in LiP from *P*. *chrysosporium*, a surface-exposed tryptophan is reported to be the interaction site for VA^113^.

### Biotechnological applications of DypB

Despite the identification of many ligninolytic enzymes such as LiPs, MnPs and laccases in fungi, they have not been used for commercial production of ligninolytic enzymes owing to the inherent complexity of fungal genetics and challenges of their protein expression^38,45,112^. Bacterial ligninolytic enzymes circumvent these limitations and offer great potential for biotechnological applications including lignocellulosic biomass conversion to biofuels, biopulping and biobleaching in paper industries, food industries, bioremediation of phenolic compounds etc.^112,114^. Furthermore, bacteria have the ability to survive under different environments, and hence, their enzymes may possess wider range of activities, pH and thermal stabilities relative to fungal lignin-degrading enzymes^115^. The potential of the DyP-type peroxidases in wide-ranging applications is revealed by their ability to degrade synthetic dyes^111^ and delignification of recalcitrance biomass^38,45,106^. Our findings revealed that accumulation of DypB in transgenic tobacco increased glucose yield by as much as 91% as compared to the non-transgenic control (248 mg/g vs 130 mg g^−1^ DM) which appears to be due to modification of lignin *in planta* prior to saccharification. To our knowledge, this is the first report showing an increase in saccharification efficiency by producing and activating a lignin-degrading enzyme *in planta* without any pretreatment, revealing the potential of the DypB in breaking recalcitrance and facilitating the conversion of liginocellulosic biomass to biofuels. However, A and B-type DyPs show 100-fold lower activity than C- and D-type peroxidases^51^, suggesting the potential for incorporating the latter enzymes in future research programs addressing biomass recalcitrance through transgenic technology. Alternatively, protein engineering via directed-mutagenesis of amino acid residues as well as motif-swapping among these peroxidases can potentially produce a superior lignin degrading variant. Furthermore, given the synergistic relationship between two or more ligninolytic enzymes that has been shown to improve biomass delignification^9,116,117^, simultaneous introduction of a set of lignin-degrading enzymes into bioenergy crops may lead to an even more dramatic reduction in biomass recalcitrance. These and similar biotechnology solutions to recalcitrance would tremendously benefit the emerging lignocellulosic biomass-based biofuel and biochemical industry.

Several other important industrial applications of lignin degradation biotechnology are anticipated. Since lignin is one of the most abundant biopolymers, and is being produced in large quantities by the paper/pulp and bioethanol industries; there is a need to convert this polymer to a renewable source of high-value aromatic chemicals^10,118^. This requires efficient biocatalytic routes for lignin deconstruction using ligninolytic enzymes such as the DyP-type peroxidases as well as microorganisms with pathways engineered for the conversion of the degradation products to high value chemicals. To this end, Sainsbury *et al*.^118^ reported accumulation of vanillin (a valuable food/flavor chemical) and a small amount of ferulic acid and 4-hydroxybenzaldehyde in the ligninolytic bacteria *R*. *jostii* RHA1 in which vanillin dehydrogenase gene has been deleted, when cultured on minimal medium containing wheat straw lignocellulose and glucose. Bacterial species with even more active forms of the DyP-type peroxidases, such as *Pseudomonas fluorescens* Pf-5^96^^,^^119^ and *Amycolatopsis sp*. ATCC 39116 strains 75iv2^120^, offer a great potential for extracellular lignin degradation and intracellular aromatic catabolism as a means to valorize lignin in a single step^114^. Such ligninolytic bacteria can also be used for targeted pathway engineering for producing high-value coproducts from lignocellulose. Furthermore, *in planta* manipulation of enzymes on these pathways may prove a cost-effective way to commercialize chemicals from lignin.

Given the reduced amount of lignin derived pyrolysates from biomass in which DypB is pre-activated, such biomass may also improve the quality of bio-oil produced by fast-pyrolysis, a low-cost thermal liquefaction^121^. The use of pyrolysis-derived bio-oil as a refinery feedstock and in wider applications has been hampered by its chemical and physical properties, including several products derived from lignin such as coniferyl alcohol, sinapyl alcohol, isoeugenol, vanillin, vinylguaiacol, methyl guaiacol, guaiacol, and catechol^122,123^. Biomass pretreatment that degrades lignin has been shown to improve yield and quality of pyrolysis bio-oil^124^, and using biotechnology to enhance lignin degradation is likely to have similar effects. The approach demonstrated here at laboratory-scale, with accumulation and activation of lignin degrading enzymes such as the DypB *in planta* and subsequent removal of lignin degradation products prior to biomass pyrolysis, has scale-up potential for the production of higher grade bio-oil that can be refined into transportation fuels as well as higher value products.

## Conclusion

Current biomass pretreatment technologies are capital- and energy-intensive, making this step one of the most expensive operations in conversion of lignocellulose, and often requiring chemicals that are environmentally unsafe. Here we report a novel method of ligninolysis, whereby a bacterial lignin degrading enzyme can be accumulated in its active form, activated *in planta*, and used to improve biomass saccharification efficiency with several lines of evidence suggesting the mechanism is by depolymerizing intact lignin. This approach has the potential to tremendously reduce the cost and environmental impact of biomass pretreatment. To our knowledge this is the first report concerning the heterologous expression of a ligninase that breaks biomass recalcitrance *in planta*. Future research will explore other bacterial and fungal DyP-type peroxidases and ligninolytic enzymes for their potential application in the conversion of lignocellulosic bioenergy crops (switchgrass, *Miscanthus*, *Pennisetum purpureum*, *Populus etc*.) to biofuels. Furthermore, it is interesting to study DyP-mediated lignin modification using tetramethylammonium hydroxide (TMAH)-GC/MS thermochemolysis^125^ and *in situ* lignin modification using designer monolignols^126,127^ and a fluorogenic dye^128^ for lignin visualization, localization and quantification during lignification. To understand the potential of DyP-peroxidases, it will be important to study the compatibility of the recombinant ligninases with simultaneous saccharification and fermentation, consolidated bioprocessing, and other conversion strategies to improve the production of biofuels as well as high-value products from lignocellulose.

## Material and Methods

### Expression vector construct for transformation

To understand whether bacterial ligninases could reduce biomass recalcitrance, we investigated the dye-decolorizing lignin peroxidase DypB (Accession number ABG94212.1) from *Rodococcus jostii RHA1*). The DypB was the first bacterial enzyme intensively characterized for oxidation of lignin model compounds and polymeric lignin^45^. The coding sequence was obtained from Dr. Lindsay D. Eltis (University of British Columbia). Expression vectors were generated for the native sequence for cytosol-targeting or a sequence optimized for tobacco preferred codon (GenScript) for ER-targeting, which contained a synthetic 22 amino acid N-terminal ER-signal peptide based on Arabidopsis basic endochitinase gene (ChiB) and the C-terminal retention signal ‘HDEL’ (Fig. 1a)^81^. Since amino acid substitution (N246A), has been shown to increase catalytic activity of the enzyme *in vitro* ^54^, we introduced a single point mutation using overlap extension PCR method, involving two rounds of PCR^129^. In the first round, two PCR fragments (N and C halves) were generated using primers that introduced the mutated codon and overlapping regions on both the sense and antisense strands. In the second round, the two fragments were combined and used as templates^130^. The coding region was amplified using sense (aaggaaaggaAGATCTatgccaggcccagtcgcgagattggcaccac) and antisense (aaggaaaggaGGTACC tcagtggtgatgatgatgatgttgcgatactcctttgagaccac) primers containing *Bgl*II and *Kpn*I adapters (upper case) for subsequent cloning into the modular vector pSAT1^131^ and C-terminus polyhistidine residues; the constructs were under the control of enhanced cauliflower mosaic virus (CaMV) 35S promoter and tobacco etch virus translation enhancer. All constructs were generated by PCR amplification using Phusion Hot Start II High-Fidelity (Fisher Scientific, Pittsburgh, USA). Expression cassettes were excised at *Asc*I site and inserted into *Asc*I linearized pPZP-RCS2 binary vector containing NPTII for kanamycin resistance^132^ for subsequent transformation of Agrobacterium strain LBA4404.

### Transformation, generation and growth of transgenic lines

About 0.5 mm^2^ leaf pieces of 4–6-week-old *Nicotiana benthamiana* plants were infiltrated with agrobacterium harboring the expression vectors pPZP-NPTII-DypB_cyto_, pPZP-NPTII-DypB_ER,_ pPZP-NPTII-DypB_N246A_ or the empty vector pPZP-NPTII for five minutes in the presence of 200 μM acetosyringone. The explants were blotted on sterile filter paper to get rid of excess agrobacterium before they were transferred to tobacco shoot induction medium (TSM) containing 1 mg l^−1^ cytokinin (BAP) and 0.1 mg L^−1^ auxin (NAA) for four days. The explants were washed thoroughly with sterile water containing 250 mg L^−1^ carbenicillin. The explants were then incubated on TSM medium containing 100 mg L^−1^ Kanamycin and 250 mg L^−1^ Carbenicillin. After 3–5 weeks, putative transgenic shoot primordia emerged, which were allowed to elongate until the shoots were separated from the callus and transplanted into tobacco modified root initiation medium (Phytotechnology laboratories, Shawnee Mission, USA). Putative transgenic *in vitro* plantlets were transplanted into 4-inch square pots containing Sta Green potting mix (Lowe’s, Dover, DE) and grown in the greenhouse under natural light for about three months.

### Verification of transgene insertion

To verify transgene insertion, genomic DNA was isolated from 100 mg of fresh leaves of wild type or transgenic lines using the GeneJET Plant Genomic DNA Purification Mini Kit (Thermo Scientific) according to manufacturer’s instructions. The DNA was used as a template in PCR reactions to amplify the DypB gene. The coding region of the native DypB sequence was amplified using sense (5′-atgccaggcccagtcgcgagattggc-3′) and antisense (5′-gtggtgatgatgatgatgttgcgatac-3′) primers while for the optimized sequence; sense (5′-atgaaaactaatcttttcttgtttcttatc-3′) and antisense (5′-tcacaactcgtcatgatgatgatgatg-3′) primers were used. The selectable marker gene neomycin phosphotransferase (NPTII) was amplified by a sense (5′-atggggattgaacaagatggattgc-3′) and antisense (5′-gaagaactcgtcaagaaggcgatag-3′) primers. The PCR products were analyzed by agarose gel electrophoresis, stained in ethidium bromide and visualized under ultraviolet light.

### RNA extraction

Total RNA was extracted from five individual lines of each cytosol targeting (Cyto2, Cyto3, Cyto4, Cyto7 and Cyto8), ER-targeting (ER1, ER2, ER5, ER7 and ER10), and ER-targeting mutant Asn246Ala (N246A1, N246A2, N246A4, N246A7 and N246A10). Fully expanded leaves (third from top) of two-month-old plants were immediately frozen in liquid N_2_ and ground to a fine powder using a mortar and pestle. Total RNA was isolated using the RNeasy Plant Mini Kit (Qiagen, Valencia, CA) according to the manufacturer’s instructions. The RNA solution was stored at −80 °C until subsequent use.

### Quantitative real-time RT-PCR

To determine the level of DypB transcripts in *N*. *benthamiana*, we performed quantitative real-time RT-PCR (pPCR) using an ABI 7500 real-time PCR system and SYBR Green Kit (Applied Biosystems, Grand Island, USA). Total RNA was treated with DNase I (Invitrogen, Carlsbad USA) to remove contaminating genomic DNA prior to first-strand cDNA synthesis. One microgram of the first-strand cDNA was used for each (pPCR) reaction containing 2xPower SYBR Green Master Mix and 0.15 µM primers in a final volume of 25 µL. Gene-specific sense (5′-CAGTTACGTGATCGTGCAG-3′) and antisense (5′-GAGGGTGACGTGCGAATTGC-3′) for native (cytosolic-targeting), and sense (5′-GACCAGTGCATAGTGCACC-3′) and antisense (5′-CATGCACTTCATCCACAACTG-3′) for optimized (ER-targeting) sequences were used to amplify the DypB gene. An internal control (18S) RNA was amplified by sense (5′-CGCAAGACCGAAACTCAAAG-3′) and antisense (5′-TGTTCATATGTCAAGGGCTG-3′) primers. Relative expression levels were calculated from two biological, and four technical replicates using the ΔΔC_T_ method available on SDS software (Applied Biosystems).

### Cellular localization of EGFP-DypB constructs

Enhanced green fluorescent protein (EGFP)-fusion constructs were generated by sub-cloning the DypB sequences with or without the ER-targeting and retention signal peptides into pSAT-1491 vector containing N-terminal EGFP tagging^133^ under the control of enhanced CaMV 35s promoter. Expression cassettes were restriction digested using *Asc*I and assembled into pPZP-NPTII binary vector. The binary vector was introduced into Agrobacterium strain GV3101 for transient expression in tobacco leaves. Agrobacterium carrying the constructs were grown overnight at 28 °C in the presence of rifampicin (30 mg L^−1^) and spectinomycin (100 mg L^−1^) appropriate antibiotics and acetosyringone (100 mM). After overnight culture and OD600 of 1.0–1.2, the culture was pelleted and washed with sterile infiltration media containing 10 mM MOPs and 10 mM MgCl_2_ (pH 5.5) and 200 mM acetosyringone. The OD600 was adjusted to 0.4 and the suspension was incubated at 28 °C for 1 h before it was used to infiltrate five-week old tobacco the leaves from the abaxial side using 1 ml syringes. Leaves were also infiltrated with *Agrobacterium* carrying positive controls pPZP-NPTII-EGFP and CD3-955^57^ for cytosol and ER-targeting, respectively. Empty vector pPZP-NPTII was used as negative control. Infiltrated plants were incubated at 28 °C in a controlled growth chamber. After four days sample of leaf sections (~0.4 mm × 10 mm) were taken and mounted on microscope slides with a drop of ProLong Gold antifade reagent (Fisher) and covered with a cover slide. The specimen was stored at 4 °C overnight prior to imaging using inverted Olympus Fluorview 10i confocal laser scanning microscope (http://www.olympus-lifescience.com/en/laser-scanning/) at excitation and emission wavelengths of 588 and 499–520 nm, respectively.

### Recombinant protein purification, SDS-PAGE and western blot

For protein extraction, fully expanded leaves from the top part of two-month-old wild type or the transgenic lines were ground to a fine powder under liquid N_2_ using mortar and pestle, and homogenized using CelLytic P reagent (Sigma, St. Louis, USA). The homogenate was filtered through four layers of mira cloth into a 50-mL tube and centrifuged at 12,000 × g at 4 °C to pellet the leaf debris. The supernatant was recovered for subsequent protein purification and downstream assays. Protein concentration was determined using the Coomassie (Bradford) Protein Assay Kit (Thermo Scientific, Waltham, USA) using BSA as a standard according to the manufacturer’s protocol. The DypB protein was purified using HIS-Select Nickel Affinity Gel (Sigma), which is an immobilized metal ion affinity chromatography (IMAC), charged with the nickel ions that are designed to specifically bind histidine containing proteins. The affinity gel was equilibrated with 50 mM sodium phosphate buffer (pH 8.0) containing 300 mM NaCl. After centrifuging at 5000 × g for 5 min, the equilibration buffer was removed, and 2 mg of total protein form DypB-ER and 10 mg from DypB-Cyto was loaded onto the affinity gel, and allowed to bind while being agitated on a rotary shaker (~175 rpm) at 4 °C for 2 h. For the cytosol constructs, the starting amount of total protein was increased because expression level was lower than that of the ER constructs. The samples were centrifuged at 5000 × g for 5 min, and the supernatant was discarded. The affinity gel was washed five times with the equilibration buffer. The gel was then suspended in elution buffer (50 mM sodium phosphate (pH 8.0) containing 0.3 M sodium chloride, and 100 mM imidazole, and the protein was eluted by gentle shaking for 20 min; this was repeated at least three times, and the fractions were recovered. The eluate (300 μL) was loaded onto the dialysis tubes, and dialyzed against 20 mM sodium acetate buffer using Pur–A–Lyzer protein dialysis kits (Sigma) with 12–14 kDa molecular weight cut–off for 6 h with dialysis buffer replaced after 3 h. To determine the level of DypB protein, 10 μL of purified protein sample from DypB-ER and 20 μL from DypB-Cyto was loaded onto 12% polyacrylamide gel along with controls and separated by electrophoresis. The proteins on the gel was transferred to Amersham™ Hybond PVDF membrane (GE Healthcare Piscataway, USA) and probed with mouse Anti-His Tag Monoclonal Antibody (Lifetien, South Plainfield, USA). A donkey polyclonal antibody to mouse IgG (Abcam Inc, Cambridge, USA) was used as a secondary antibody. Color development was obtained by adding a combination of NBT/BCIP (nitro-blue tetrazolium chloride and 5-bromo-4-chloro-3′-indolyphosphate p-toluidine) salts substrate solution directly onto the membrane. DypB protein in Cyto lines was detected using PloyExpress Gold anti-DypB antibody raised in rabbits (GeneScript, Piscataway, USA), and a donkey polyclonal anti-rabbit IgG (Abcam Inc) was used as a secondary antibody.

### Enzyme activity assay

Enzyme activity was determined using the standard peroxidase substrates, DMP and the chromogenic ABTS, as well as lignin-related substrates Kraft lignin and VA. For ABTS and DMP the assay was performed spectrophotometrically according to Herter *et al*.^134^ and Roberts *et al*.^65^ with modifications. A total volume of 1.5 mL contained 1225 μL of 50 mM Na-acetate pH (5.5), 37.5 μL of 100 mM MnCl_2_, 37.5 μL of 40 mM H_2_O_2_, 50 μL (5–15 μg) of DypB enzyme, and 150 μL of 100 mM ABTS or 15 μL of 50 mM DMP. The activity of DypB enzyme in oxidizing Kraft lignin was assayed according to Ahmad *et al*.^45^ with slight modification. The assay was performed in 1 mL reaction containing 500 μL of 0.5 mg mL^−1^ Kraft lignin stock, 175 μL of 50 mM succinate buffer (pH 5.5), 200 μL of 50 mM MnCl_2_, 50 μL (5–15 μg) of DypB enzyme, and 100 μL of 40 mM hydrogen peroxide. The molar concentration of Kraft lignin was calculated based on average molecular weight of 10,000 g mol^−1^. The rate of oxidation of VA was measured according to Sarkanen *et al*.^134^ with modifications. The assay mixture (1.5 mL) consisted of 500 μL of 3 mM VA solution (prepared in 0.33 M sodium tartrate buffer (pH 3.0)), 15 μL of 100 mM MnCl_2_, and 37.5 μL of freshly prepared 40 mM H_2_O_2_. Because heme co-factor has been shown to enhance DyP enzyme activity^135^, recombinant enzyme was reconstituted using hemin according to Min *et al*.^49^. Briefly, 10 μL of hemin solution (10 mg mL^−1^) dissolved in dimethylsulfoxide was added to 1 mL of purified DypB and then incubated on ice for 1 hour while shaking at 100 rpm. The protein samples were centrifuged at 5,000 × g for 5 min to remove excess hemin, and the supernatant was used for determining VA oxidizing activity of the recombinant protein. For all the substrates, reactions were initiated with the addition of H_2_O_2_. Enzyme specific activities were equivalent to micromoles of each substrate oxidized, based on increase in absorbance per min monitored spectrophotometrically at λ = 468 nm (ABTS), 420 nm (DMP), 465 nm (Kraft lignin) and 310 nm (VA), and extinction coefficients of 49,600, 36,000, 10018 and 9300 M^−1^ cm^−1^ at each wave length, respectively. Reactions were initiated by adding the H_2_O_2_. The final absorbance was corrected for the nonenzymatic reaction with H_2_O_2_ for DMP, Kraft lignin and VA.

### Biomass saccharification and sugar analysis

Mature tissue (stems, leaves, inflorescence and seeds was expected to have sufficient DypB enzyme) was sampled from the top 25% of the shoot of three months old, greenhouse established T1-transgenic or non-transgenic plants. This tissue was chopped to about 1 cm pieces and immediately frozen in liquid N_2_, grounded to a fine powder using mortar and pestle, and stored in the freezer (−80 °C) until use. One gram of the frozen biomass was weighed into a 50 mL Falcon tubes containing 9 mL of DypB assay buffer (50 mM sodium acetate pH 5.5 containing 5 mM MnCl_2_ and 2 mM H_2_O_2_)^45^, and the samples were incubated for three days at room temperature while shaking at 250 rpm. The samples were then centrifuged at 9000 g for 10 min, and the biomass was washed twice with the DypB assay buffer to remove lignin-derived compounds, which may inhibit cellulase activity^136,137^, and to also remove free sugars released from the biomass, which can interfere with the determination of the actual amount of sugar released during saccharification. The final pellet was suspended in 9 mL of 50 mM sodium acetate pH 5.0 to which 100 μL of each *Trichoderma reesei* cellulase and *Aspergillus niger* glucosidase (Sigma)^138^, and 200 μL sodium azide (from 2% stock) to prevent microbial growth^62^. Control samples without the enzymes were also prepared for each treatment. The reactions were then incubated at 45 °C for three days while shaking at 250 rpm. After three days, the samples were centrifuged at 10,000 g for 5 min and the resulting clear hydrolysate was filtered (0.22 μm) for sugar analysis. The monosaccharides (glucose, galactose, mannose, xylose and arabinose) in the hydrolysate were determined using Dionex-Ion Exchange Chromatography 3000 equipped with an electrochemical detector (Dionex, Sunnyvale, USA). Samples were separated using a CarboPac PA20 with pulsed amperometry (3 mm × 30 mm) and an analytical (3 mm × 150 mm) columns at a flow rate of 0.5 mL/min and column temperature of 30 °C. The samples were eluted with 2 mM NaOH. The column was flushed with 200 mM NaOH and 18 MΩ water between each sample. The amount of sugars released was calculated from the peak areas after calibration with standards of known concentration for each sugar species. The sugar concentration from the IC reading was converted to milligram of sugar per gram dry matter. The dry matter content of the tissue samples was 28% of fresh weight, determine by an overnight incubation at 105 °C to constant weight. Sugar yield was measured from at least four replicates for each treatment.

### Biomass pyrolysis-GC-MS

Biomass, including stem, leaf, inflorescence and seeds obtained from the top 25% of the shoot, was ground at liquid nitrogen temperature using a mortar and pestle. The resulting powder was dried overnight at room temperature in a vacuum oven. One half of each sample was directly subjected to pyrolysis, while the other half was treated for *in planta* DypB activation as follows: approximately 100 mg of the dried biomass was placed in a scintillation vial with 650 μL of 50 mM succinate buffer, 25 μL of 40 mM H_2_O_2_, and 100 μL of 100 mM MnCl_2_ added. The reaction mixture was incubated at 30 °C for 24 h while being agitated on a stirrer plate. The samples were transferred to a centrifuge tubes containing 50 mL deionized H_2_O and centrifuged at 2500 rpm for 30 min. The supernatant was decanted and the pellet was dried as stated above prior to pyrolysis. Pyrolysis was performed as described by Harman-Ware *et al*.^60^. In brief, a Pyroprobe Model 5200 (CDS Analytical, Inc., Oxford, USA) was used, coupled to an Agilent 7890 GC equipped with an Agilent 5975C MS detector. The pyroprobe was run in direct mode under an atmosphere of He. Pyrolysis was performed at 650 °C for 20 s (heating rate of 1,000 °C/s). The GC oven and transfer lines were maintained at a temperature of 325 °C. A DB1701 column was used (60 m ×0.25 mm ×0.25 μm), the temperature program for the GC being as follows: 45 °C for 3 min, followed by ramping to 280 °C at 4 °C/min and a 10 min hold. A flow rate of 1 mL/min was used, employing He as the carrier gas. Inlet and auxiliary lines were held at 300 °C, the MS source being set at 70 eV. One milligram of ground biomass sample was analyzed in a quartz cell, quartz wool being used to hold the sample in place. Samples were heated to 100 °C for 10 s in the probe prior to analysis in order to remove any residual water. Prior to sample analysis, blank experiments were performed to validate the cleanliness of the system. After sample analysis, methanol was run as a sample to remove any condensed products inside the pyroprobe. Methanol and blank experiments were repeated as necessary until the system was clean.

### Biomass composition analysis

We assessed the biomass composition using two independent procedures to determine whether there was any considerable variation in the initial biomass content prior to saccharification or pyrolysis. The first method was based on NREL procedures^62,63^. Biomass was dried at room temperature until moisture content was below 10%. Dry biomass was rinsed three times with 100 °C water followed by three rinses of ethanol also at 100 °C using a Dionex accelerated solvent extractor to separate nonstructural components (water soluble inorganics, non-structural sugars, nitrogenous materials and ethanol soluble chlorophyll and waxes) from structural components such as cellulose, hemicellulose and lignin. A subsample of the water extract was analyzed for sucrose and glucose and the remainder was dried under vacuum and weighed. The ethanol extract was also dried under vacuum and weighed. The extractives free biomass was then hydrolyzed using 72% H_2_SO_4_ followed by 4% H_2_SO_4_ in an autoclave. After autoclaving, the hydrolysates were filtered and the filtrate was analyzed for monosaccharides. Glucan content was calculated from glucose concentration after adjusting for starch content in the extractive free biomass. The remaining solid fraction was dried and weighed for acid insoluble lignin. Acid soluble lignin was quantified spectrophotometrically. The second biomass composition analysis was performed according to van Soest^64^. Briefly, the biomass was dried at 60 °C for 4 h and ground to 1 mm size. Part of the biomass (0.5 g) was incubated with neutral detergent fiber solution containing α-amylase and sodium sulfite for 75 min, rinsed twice with α-amylase and finally soaked in acetone for 3 min to obtain Neutral detergent fiber (NDF). Another 0.5 g biomass was incubated in acid detergent fiber solution containing 72% sulfuric acid at 110 °C for 4 h, rinsed three times with boiling water and soaked in acetone for 3 min to obtain acid detergent fiber (ADF). The ADF was further incubated in 72% sulfuric acid at 20 °C and the insoluble fraction without ash was used for lignin determination. Ashing was done at 550 °C for 3 h. Hemicellulose content was determined by subtracting ADF from NDF, and the mass of cellulose was calculated from ADF by subtracting the mass of lignin and ash.

### Statistical analysis

For each genetic construct, at least 10 transgenic T_0_ lines were generated, and five randomly selected lines were considered for phenotypic and genotypic analysis. Gene expression level was determined from two biological and four technical replicates of transgenic and control lines. The amount of sugars released after saccharification was analyzed from four biological replicates per construct. Biomass composition and Pyrolysis-GC/MS analysis was conducted on duplicate samples per line. Standard errors were calculated using Microsoft Excel 2010. Sugar data was analyzed using one-way ANOVA using the PROC GLM procedure in SAS^139^. After significant F-tests, the Tukey multiple comparison procedure was used to separate the means (p < 0.05).

### Data Availability

All relevant data are available from the corresponding author upon request.

## Electronic supplementary material

Supplementary Tables and Figures
