# Supplementary material for: Reducing biomass recalcitrance by heterologous expression of a bacterial peroxidase in tobacco (Nicotiana benthamiana)
Source: Sci Rep. 2017 Dec 6;7:17104. doi: 10.1038/s41598-017-16909-x (PMC5719049; doi:10.1038/s41598-017-16909-x)
Supplement: Supplementary file 1 — Supplementary Tables and Figures [file 41598_2017_16909_MOESM1_ESM.pdf]

Supplementary Tables and Figures

Reducing biomass recalcitrance by heterologous expression of a bacterial peroxidase in tobacco (*Nicotiana benthamiana*)

Ayalew Ligaba-Osena[1], Bertrand Hankoua[1*], Kay DiMarco[2], Robert Pace[3], Mark Crocker[3], Jesse McAtee[4], Nivedita Nagachar[5], Ming Tien[5], Tom L. Richard[2]

[1]College of Agriculture and Related Sciences, Delaware State University, 1200 N DuPont Highway, Dover, DE 19901, USA

[2]Agricultural and Biological Engineering, Pennsylvania State University, 111 Research Unit A, University Park, PA 16802, USA

[3]Center for Applied Energy Research, University of Kentucky, 2540 Research Park Drive, Lexington, KY 40511, USA

[4]Department of Chemistry and Biochemistry, University of Delaware, Newark, DE 19716, USA

[5]Department of Biochemistry and Molecular Biology, Pennsylvania State University, 305 South Frear Laboratory, University Park, PA 16802, USA

*Corresponding author: bhankoua@desu.edu

Other authors: AL (aosena@desu.edu), KD (kmd5507@engr.psu.edu), RP (robert.pace@uky.edu), MK (mark.crocker@uky.edu), JM (jesse.mcatee@jmusa.com), NN (Nun3@psu.edu) , MT (mxt3@psu.edu), TLR (tlr20@psu.edu)

Supplementary Table S1. Composition of biomass saccharification products as determined by ion chromatography. The recombinant DypB enzyme was activated *in situ* and the biomass was subjected to saccharification by a cocktail of cellulase and glucosidase. The amount of sugars released in the hydrolysate was determined based on standards of known concentration for each sugar species using IC-Dionex as described in the Materials and Methods. Values are means and standard error (n = 4)

Line	glucose	xylose	mannose	arabinose	Galactose
wt	130±2.0	4.5±0.1	5.8±0.1	1.8±0.0	0.6±0.0
PTII	121±0.4	4.7±0.0	5.3±0.0	1.8±0.0	0.6±2.1
cyto2	178±1.0	6.9±0.0	3.5±0.1	1.8±0.0	1.4±0.2
Cyto3	129±4.8	5.6±0.1	5.4±2.0	1.0±0.1	0.4±0.1
Cyto4	125±2.6	5.5±0.2	3.2±0.0	1.4±0.1	0.6±0.1
Cyto7	248±1.6	4.4±0.1	1.8±0.0	1.3±0.0	1.7±0.0
Cyto8	202±0.8	6.1±0.0	2.3±0.0	1.5±0.0	1.6±0.0
ER1	172±0.9	5.3±0.0	2.1±0.0	1.5±0.0	1.1±0.0
ER2	191±4.0	6.2±1.8	5.4±2.0	1.8±0.1	1.9±0.1
ER5	189±0.5	9.1±0.0	3.2±0.0	1.8±0.0	1.4±0.0
ER7	193±1.1	4.7±1.6	3.9±1.4	2.9±0.1	1.1±0.0
ER10	171±1.5	3.8±0.0	6.4±0.0	2.1±0.0	1.6±0.0
N246A1	116±0.8	11.9±0.1	5.7±0.1	4.1±0.1	4.3±0.0
N246A2	163±1.2	5.3±0.1	5.0±0.1	1.9±0.0	1.8±0.1
N246A4	153±0.6	4.7±0.0	5.5±0.1	1.9±0.1	0.8±0.0
N246A7	144±0.8	4.1±0.0	5.8±0.1	2.0±0.1	0.8±0.0
N246A10	187±0.3	5.6±0.0	3.9±0.0	1.8±0.0	1.3±0.0

Supplementary Table S2. Biomass composition analysis following NREL procedures[60,61]. The analysis was performed as described in the Materials and Methods. Values represent mean percent of total biomass dry matter ± standard errors (n=2)

Line	Structural Inorganics	Soil	Sucrose	Starch	Water Extractable	Ethanol Extractives	Lignin	Glucan	Xylan	Galactan	Arabinan	Mannan	Acetyl	Total mass
WT	2.3±0.5	8.3±0.0	7.1±0.1	1.80	22.4±0.4	4.8±0.2	8.1±0.0	19.7±0.8	7.8±0.6	1.1±0.0	0.7±0.0	1.6±0.0	4.8±0.2	91.0±1.4
NPT II	2.8±0.1	7.9±0.2	11.0±0.3	2.50	21.6±0.5	5.0±0.1	7.1±0.1	19.3±1.4	7.5±0.7	1.1±0.1	0.7±0.1	1.5±0.1	4.0±1.1	92.2±3.1
Cyto7	1.9±0.0	8.8±0.1	6.9±0.1	1.90	27.7±4.9	4.9±0.0	7.0±0.0	16.0±1.3	6.6±1.9	0.8±0.1	0.7±0.3	1.2±0.0	3.8±0.4	90.2±0.0
Cyto8	3.2±0.3	7.5±0.3	8.1±0.2	2.50	22.9±1.2	4.9±0.1	7.7±0.2	20.4±1.0	7.8±0.5	1.1±0.1	0.7±0.0	1.5±0.1	4.0±1.4	91.4±2.5
N246AL1	1.8±0.1	9.7±0.3	6.2±0.1	1.40	28.8±5.3	4.9±0.0	7.2±0.8	15.5±0.6	6. 9±1.0	1.0±0.1	0.6±0.1	1.5±0.3	4.3±1.0	90.4±1.2
N246AL10	2.9±0.4	8.9±0.4	7.0±0.4	2.00	23.2±0.6	5.0±0.4	7.9±0.1	20.2±0.3	8.1±0.2	1.0±0.1	0.7±0.1	1.7±0.0	4.6±0.3	92.7±1.7

Supplementary table 3. Biomass composition analysis using the Van Soest[62] method. Analysis was performed as described in the Materials and Methods. ***** Acid Detergent Fiber (**ADF)** is the residue remaining after incubating the biomass in detergent solution containing H_2SO_4. Neutral Detergent Fiber (a**NDF)** is the residue remaining after incubating the biomass in detergent solution containing α-amylase and sodium sulfite. Values represent mean percent of total biomass dry matter ± standard errors (n=2)

Line	ADF*****	aNDF*	starch	Lignin	ASH	Non-structural	cellulose	Hemicellulose
WT	34.90±0.0	45.00±0.0	2.00±0.1	3.30±0.0	10.24±0.0	55.00±0.0	21.36±0.0	10.10±0.0
NPTII	33.45±0.1	41.15±0.3	2.50±0.0	3.25±0.2	10.30±0.2	58.85±0.3	19.90±0.4	7.70±0.2
CYTO7	34.65±0.8	43.35±0.4	1.85±0.0	3.35±0.4	10.34±0.1	56.65±0.4	20.96±1.2	8.70±1.1
CYTO8	36.25±0.5	44.55±0.3	2.50±0.0	3.70±0.4	10.49±0.0	55.45±0.3	22.06±0.8	8.30±0.8
N246A1	34.20±0.0	43.00±0.1	1.35±0.1	3.30±0.2	11.82±0.1	57.00±0.8	19.08±0.3	8.80±0.8
N246 A10	37.90±0.7	45.60±0.4	1.50±0.1	4.20±0.0	11.53±0.0	54.40±0.4	22.17±0.7	7.70±1.1

Supplementary Fig. S1. **Supplementary Fig. S1**. a) Performance of 3-week-old seedlings of representative transgenic and wt tobacco lines on ½ MS medium with or without 50 mg/L Kanamycin. Note that the growth of Cyto7 is slower than the other transgenic lines only on the selection medium which could be due to lower activity of the aminoglycoside 3'-phosphotransferase (*aph* (3')-II or NPTII) enzyme. One hundred sterilized seeds were germinated per plate. b) PCR confirmation of DypB insertion in to *N. benthamiana* genome using genomic DNA as a template from four independent lines. A pair of gene-specific primers was used to amplify DypB (upper bands) and the selectable marker NPTII (lower bands).

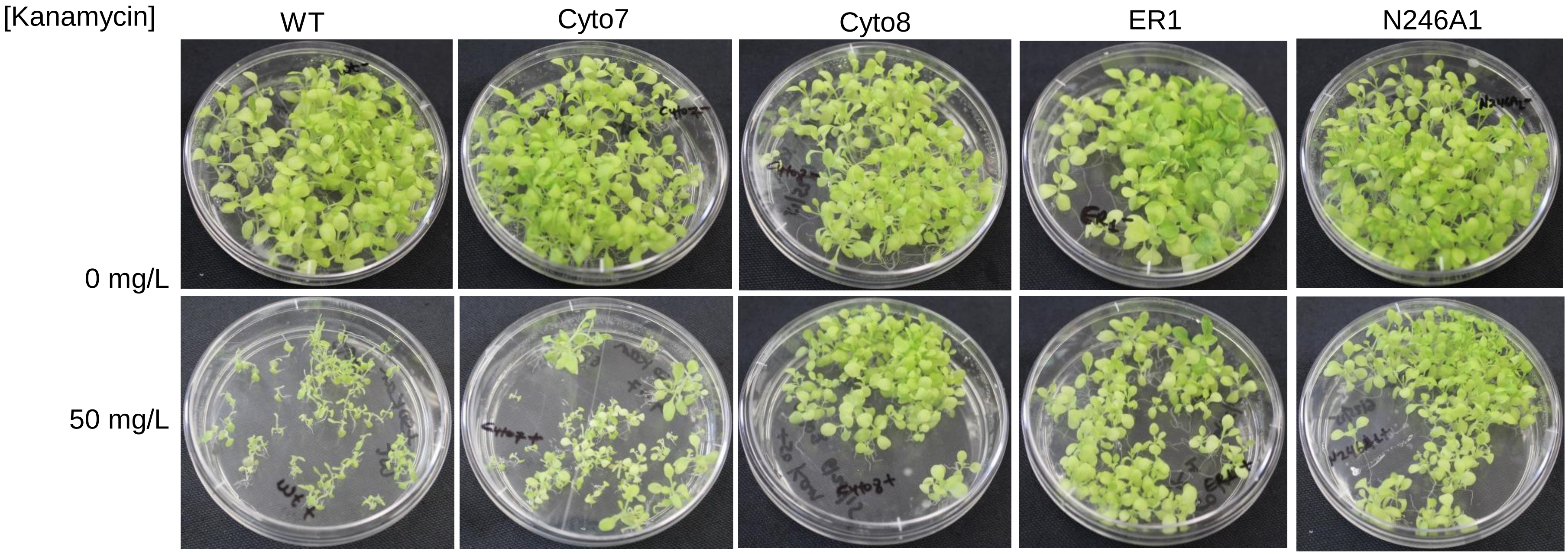

Supplementary Fig. S2. **Supplementary Fig. S2**. Cellular localization of the N-terminal EGFP constructs. The DypB sequence with or without ER-targeting and retention signal peptides were expressing in tobacco leaves along with respective controls. Expression of cytoplasmic EGFP (a), EGFP::DypB-Cyto (b), EGFP::DypB-ER (c), and the ER-marker CD3-955 (d). The corresponding bright-field images are shown in (a’) - (d’).

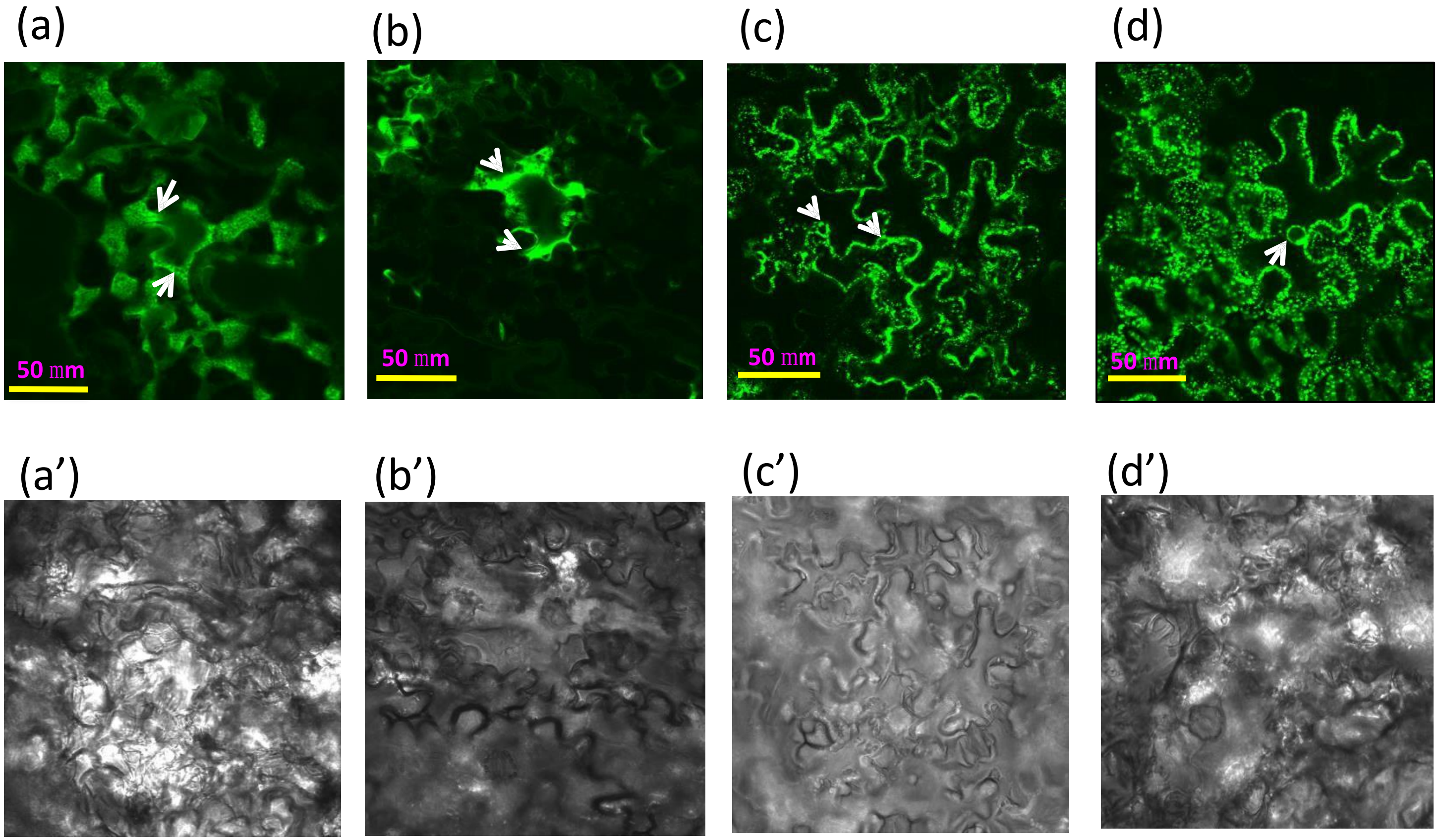

Supplementary Fig. S3. **Supplementary Fig. S3.** Comparison of a pyrograms from WT and Cyto. The DypB enzyme was preactivated *in situ* prior to pyrolysis. Pyrolysis was performed at 650°C as described in the 'Materials and Methods'. A representative pyrogram from WT (red) and Cyto (blue) are presented.

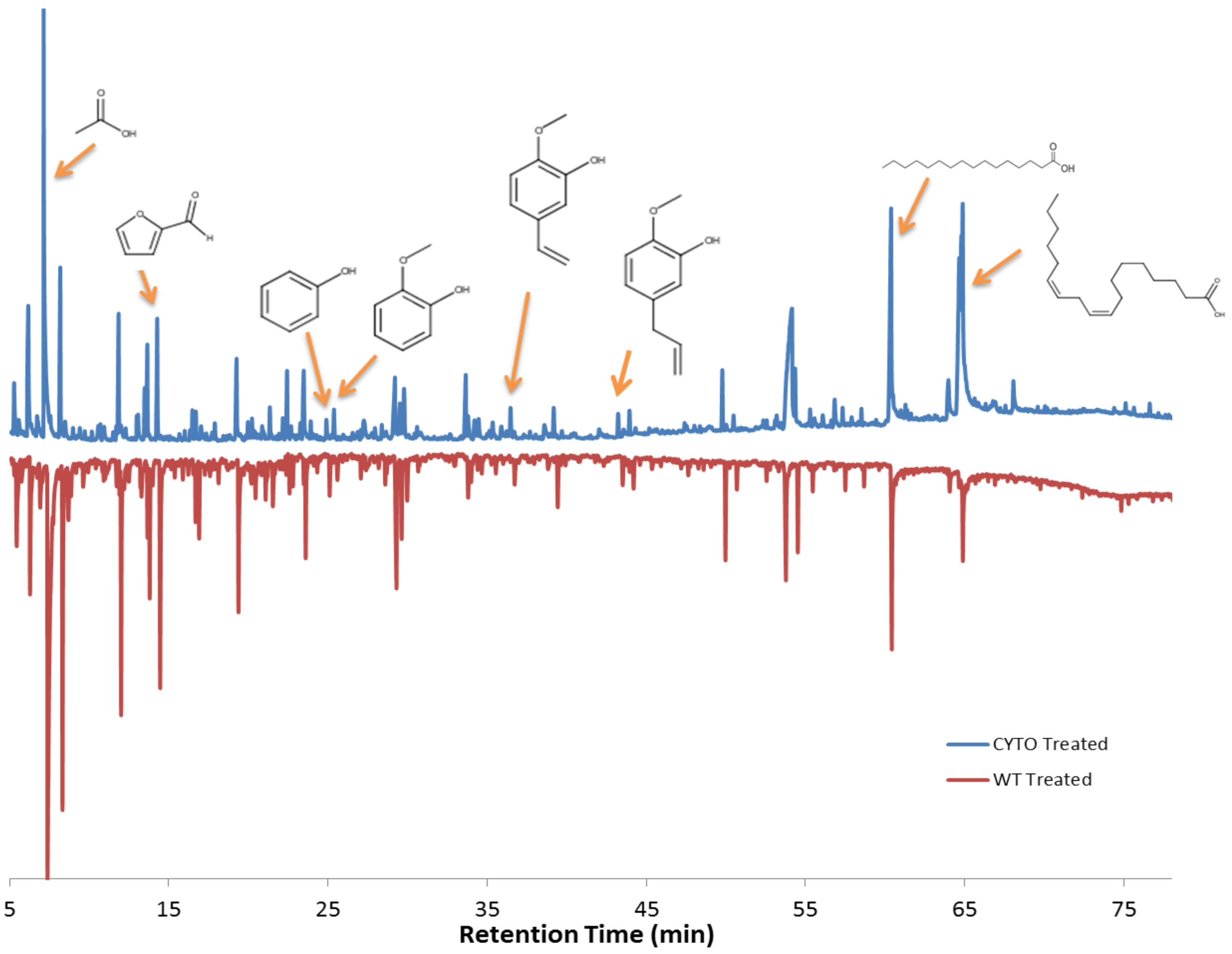

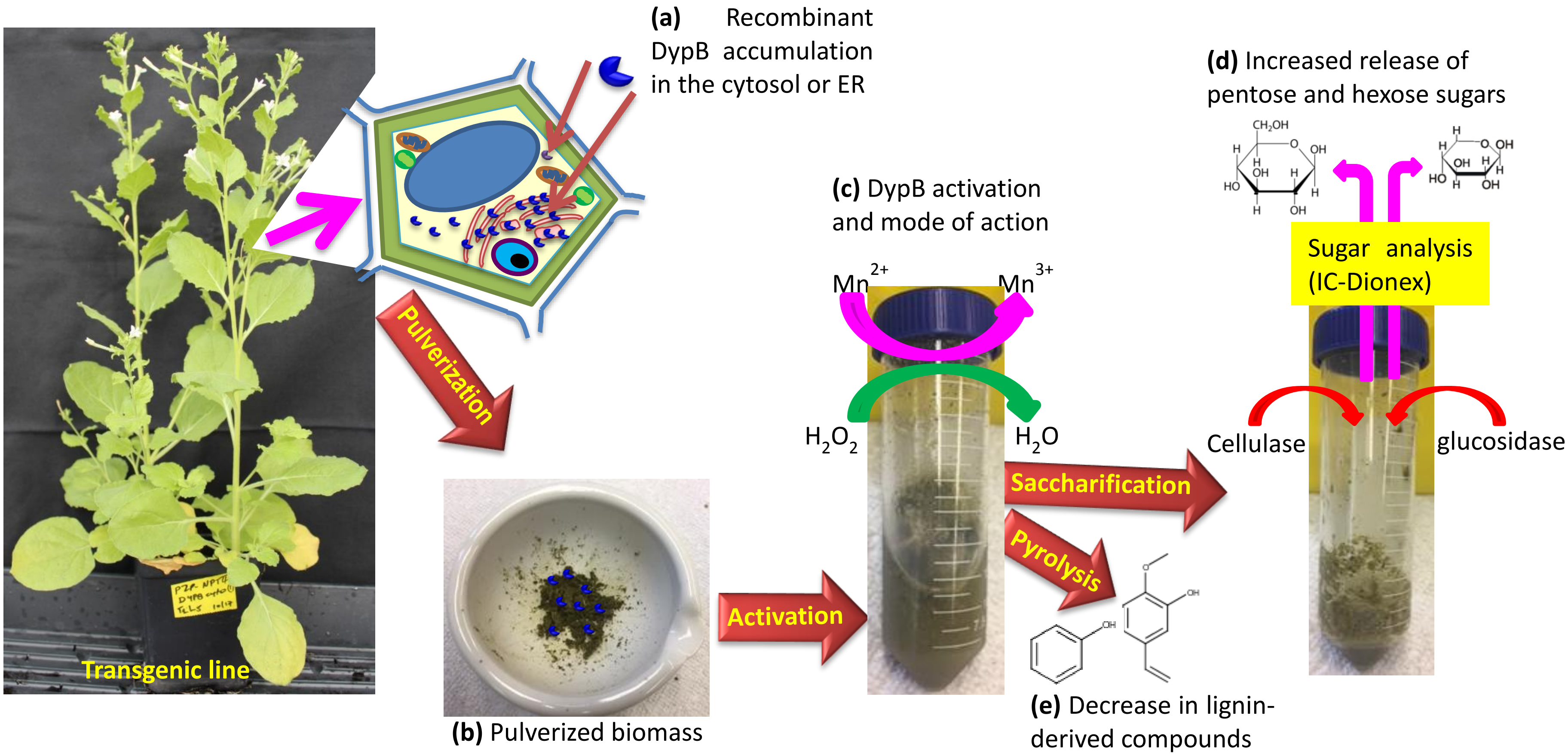

Supplemental Fig. 4. A model summarizing heterologous expression of *Rodococcus jostii DypB* in transgenic *N. benthamiana for* lignin self-depolymerization and enhanced biomass saccharification efficiency. **(a)** Recombinant DypB was accumulated in the cytosol or ER. **(b)** The biomass was pulverized under liquid N_2 to release the recombinant DypB from cellular compartments. **(c)** DypB activation in the presence of $MnCl_2$ and H_2O_2 and mode of lignin depolymerization. **(d)** biomass saccharification to fermentable sugars by the action of fungal cellulases and glucosidase enzymes. (e) Pyrolysis-GC/Ms analysis for identification and quantification of lignin-derived compounds. NB: Simplified picture of plant cell (**a**) is drawn by A L-O.
